# Photodegradation Study of Sertindole by UHPLC-ESI-Q-TOF and Influence of Some Metal Oxide Excipients on the Degradation Process

**DOI:** 10.3390/pharmaceutics11070299

**Published:** 2019-06-27

**Authors:** Jakub Trawiński, Robert Skibiński

**Affiliations:** Department of Medicinal Chemistry, Faculty of Pharmacy, Medical University of Lublin, Jaczewskiego 4, 20-090 Lublin, Poland

**Keywords:** sertindole, iron oxides, titanium dioxide, photodegradation, photocatalysis, PCA

## Abstract

The evaluation of the influence of the excipients present in the pharmaceutical formulations on the drug stability is an important part of quality control of medicines. One of the most commonly applied group of excipients are pigments, such as titanium dioxide or various forms of iron oxides, which are well-known photocatalytic agents. Therefore, the photostability of an atypical antipsychotic drug sertindole and the influence of pigments commonly used in the pharmaceutical formulations (FeOOH, Fe_2_O_3_, and TiO_2_) on this process were studied. The quantitative and qualitative analysis of the process was performed with the use of ultra high pressure liquid chromatography with diode array detection (UHPLC-DAD) system coupled with a high resolution hybrid electrospray ionization quadrupole time-of-flight (ESI-Q-TOF) mass spectrometer. Sertindole turned out to be a highly photolabile molecule. Overall 18 transformation products were found, mainly formed as a consequence of dechlorination, hydroxylation, and dehydrogenation. In all the experiments, except the TiO_2_-mediated photocatalysis, the product of chlorine substitution with a hydroxyl group was the major product. The presence of Fe_2_O_3_ and TiO_2_ accelerated the degradation process, while FeOOH served as its inhibitor. The experiments conducted with the use of the pharmaceutical formulations confirmed the catalytic activity of the used excipients. The exploration of the obtained phototransformation profiles with the use of principal component analysis (PCA) revealed that the presence of both iron oxides could influence the qualitative and quantitative aspect of the studied processes. In silico assessment of the properties showed that the transformation products are generally less toxic to rodents, possess lower hERG blocking potential, but could be more mutagenic than the parent molecule.

## 1. Introduction

Nowadays, photostability testing is an indispensable part of the drug analysis. As stated in the European Pharmacopoeia, over 250 active pharmaceutical substances (APIs) are classified as photolabile molecules and their exposure to light should be avoided [1]. The consequences of such behavior could be divided into two groups. First, the interaction of APIs with UV-Vis radiation (natural or artificial) leads to their decomposition which involves the decrease or loss of the therapeutic efficiency. Second, the phototransformation products (TPs) can possess their own pharmacological activity or toxic properties significantly higher than the parent compound [2]. For that reason various guidelines regarding the photostability testing of drugs were implemented. One of them was proposed by the International Council for Harmonization of Technical Requirements for Pharmaceuticals for Human Use (ICH) as the “Stability Testing: Photostability Testing of New Drug Substances and Products” (Q1B) document [3].

The influence of metal oxides should be important from a technological point of view, relative to stability and safety, and also from an analytical point of view. One of the most commonly applied group of excipients are pigments such as titanium dioxide (white pigment) or various forms of iron oxides (yellow or red pigments). Taking into account the commonly known photocatalytic properties of such compounds [4], the assessment of their impact on the photodegradation process is particularly interesting. Although the effect of various forms of TiO_2_ addition to the pharmaceutical formulations was studied [5], the influence of the other pigmenting substances seems to be still unexplored.

Sertindole (1-[2-[4-[5-chloro-1-(4-fluorophenyl)-indol-3-yl]piperidin-1-yl]ethyl]-imidazolidin-2-one) is an atypical antipsychotic drug used for the treatment of schizophrenia and is effective both against the positive and negative symptoms of the disease [6]. The drug serves as a 5-HT2_A_, 5-HT2_C_, D_2_, and α_1_ receptors antagonist and has a selective influence on the mesolimbic dopamine neurons [7]. Such pharmacological properties are responsible for fewer extrapyramidal side effects than in the case of classical antipsychotics [8], but they can prolong the QT interval on electrocardiogram [9]. In the available literature data there are several papers describing the determination of sertindole as well as its metabolites both in the pharmaceutical formulations and biological samples using various analytical methods such as high performance liquid chromatography with ultraviolet detection (HPLC-UV) [10], fluorimetrics [11,12], or high performance liquid chromatography with mass spectrometry detection (HPLC-MS) [13]. One research paper reporting the stability-indicating methods with the use of HPLC and TLC-densitometry was also published [14], but only one TP was found and structurally characterized. Moreover, on the contrary to our observations, in the above study sertindole was found to be totally stable under the photolytic stress conditions.

The aim of the following work is to study the direct photolysis of sertindole under the simulated solar radiation, the determination of the photolysis kinetics, the identification of the TPs, *in silico* evaluation of toxicity, and chemometric analysis of the obtained results. Additionally, in order to assess the influence of pigment excipients on the phototransformation process, the effect of addition of titanium dioxide and two forms of iron oxide (yellow and red) was also evaluated. The obtained results were compared with the experiments conducted on the real sertindole pharmaceutical formulations—one containing TiO_2_ and yellow iron oxide and the other one also containing TiO_2_ and red iron oxide.

## 2. Experimental

### 2.1. Materials

Serdolect^®^ 4 mg and 16 mg tablets (H. Lundbeck A/S, Valby, Denmark), containing 4 mg and 16 mg of sertindole in each tablet, respectively, were obtained from the local pharmacy. Sertindole standard, water for liquid chromatography-mass spectrometry (LC-MS), acetonitrile for LC-MS, formic acid for LC–MS, and titanium (IV) oxide, nanopowder 21 nm particle size (Aeroxide^®^ 25) were purchased from Sigma Aldrich Co. (St. Louis, MO, USA). Water gradient grade for liquid chromatography were purchased from Merck (Darmstadt, Germany). Yellow iron oxide nanorods (FeOOH alpha, 98% 50 nm × 10 nm) and red iron oxide (Fe_2_O_3_, alpha, 98+%, 20–40 nm) were purchased from US Research Nanomaterials, Inc. (Houston, TX, USA).

### 2.2. Sample Preparation

The stock solution of sertindole was prepared in acetonitrile at concentration 0.5 mg mL^−1^ and was refrigerated at 7 °C. The working solutions were prepared by diluting the stock solution in water to obtain 10 μg mL^−1^. The catalytic suspensions were prepared by weighing an appropriate amount of each catalyst (TiO_2_, Fe_2_O_3_, and FeOOH) or a mixture of catalysts (TiO_2_–Fe_2_O_3_ and TiO_2_–FeOOH) in the volumetric flasks and adding the sertindole working solution. The obtained catalysts and catalysts mixtures loading was 100 μg mL^−1^ (in the case of mixtures 50 μg mL^−1^ the loading of each catalyst was used). The suspensions containing pharmaceutical formulations were prepared as follows: first, Serdolect^®^ 4 mg and 16 mg tablets were separately grounded in a mortar and the equivalents of 250 μg of sertindole were weighed and transferred into 25 mL volumetric flasks. Then 500 μL of the LC–MS acetonitrile was added to the flasks and after 5 min of ultrasonic sweeping the flasks were filled with ultrapure water. The obtained concentrations of sertindole and acetonitrile were on the same level as in the case of a standard solution.

### 2.3. Irradiation Procedure

For all the experiments the working solutions and suspensions were transferred into 3.5 mL quartz caped cells (l = 1 cm) mounted horizontally in Atlas Suntest CPS+ photostability chamber (Linsengericht, Germany), and irradiated simultaneously. The irradiance was set to 250 W m^−2^ which corresponds to energy dose of 900 kJ m^−2^ h^−1^. The chamber was equipped with a xenon lamp and D65 filter simulating full solar spectrum. The temperature in the chamber was controlled and kept below 35 °C. All the suspensions were vigorously stirred (500 rpm) with the use of a microstirrer (MINI Stirrer, Cimarel: Telemodul, Thermo Electron LED GmbH, Langenselbold, Germany) and polytetrafluoroethylene (PTFE) covered stirring bar (l = 6 mm) during the whole experiment. The dark control sample was also performed by exposing the working solution in quartz cell wrapped in aluminum foil for the same period of time. 100 μL solution or suspension aliquots were collected after 0, 2, 4, 8, 12, and 14 min and then centrifuged at 15,000 rpm for 5 min. The UHPLC-DAD/ESI-Q-TOF analysis was performed afterward.

### 2.4. Analytical Procedure

UHPLC-MS/MS analysis was performed using the Agilent Accurate-Mass Q-TOF LC/MS G6520B system with dual electrospray source and Infinity 1290 UHPLC system consisting of: binary pump G4220A, FC/ALS thermostat G1330B, autosampler G4226A, DAD detector G4212A, TCC G1316C module (Agilent Technologies, Santa Clara, CA, USA), and Hibar RP-18e (2.1 × 50 mm, dp = 2 μm) HR column (Merck, Darmstadt, Germany). A mixture of acetonitrile (A) and water containing 0.1% of formic acid (B) was used as a mobile phase. The gradient elution was carried out at a constant flow of 0.3 mL min^−1^ from 5% A (95% B) to 60% 0–9 min. Two-minute post time was performed to return to initial conditions. The total analysis time was 11 min. The injection volume was 1 μL and the column temperature was maintained at 35 °C. MassHunter workstation software in version B.08.00 was used for the control of the system and data acquisition.

The MS detector was tuned in a positive mode in extended dynamic range (2 GHz). To ensure accuracy in masses measurements, reference mass correction was also used and mass 121.0508 and 922.0097 (Agilent ES TOF reference mix solution) were used as lock masses. The electrospray (ESI) was chosen as an ion source. The main parameters were optimized and the following settings were applied: gas temp.: 300 °C, drying gas: 9 L/min, nebulizer pressure: 30 psig, capillary voltage: 3500 V, fragmentor voltage: 175 V, skimmer voltage: 65 V, octopole 1 RF voltage: 750 V. In order to make the qualitative and quantitative analysis in one run, data acquisition was performed in auto MS/MS mode with spectral parameters: mass range: 60–950 *m/z* and acquisition rate: 2 spectra/s (for MS and MS/MS data). Diode array detector collected data in the range 210–300 nm, and wavelength of 260 nm was selected for the quantitative analysis of sertindole. All the measurements were duplicated. For the chemometric analysis, the data acquisition was performed in the TOF mode, and six replications were done for each experiment.

Calibration of the quantitative method was performed with the use of six sertindole concentration levels (0.5, 1, 5, 10, 15, and 20 μg mL^−1^). Intraday and interday precision was evaluated using *n* = 12 and *n* = 18 replicates. The averaged retention time of sertindole was 7.103 ± 0.006 min. All that data, along with the limits of detection and quantification (LOD and LOQ) are shown in Table 1.

### 2.5. Chemometric Analysis

Six samples taken after 4 min of irradiation from each experiment were taken and submitted for LC–MS analysis. TOF (MS) mode was used for the registration of their chromatographic/spectral degradation profiles. The MFE (molecular feature extraction) algorithm from the Mass Hunter Qualitative Analysis software version B.06.00 (Agilent) was used for the data background ion noise cleaning and to extract the list of the ions characteristic for photocatalytic transformation products. The MFE parameters were optimized and the following settings were applied: maximum 1 charge state of the analyzed ions, more than 5000 counts for the compound filter, isotope model: common organic molecules with peak spacing tolerance 0.0025 *m/z*.

In order to carry out the multivariate chemometric analysis, the obtained results were next exported to the Mass Profiler Professional (MPP) software version 12.61 (Agilent Technologies Inc., Santa Clara, CA, USA and Strand Life Sciences Pvt. Ltd., Bengaluru, India). With the use of this software the data was normalized and aligned. Then the principal component analysis (PCA) was performed in order to evaluate the qualitative and quantitative differences in the registered phototransformation profiles.

### 2.6. In Silico Evaluation of Sertindole and its TPs Properties

The acute toxicity to rodents, mutagenicity and hERG inhibitory potential of the identified phototransformation products as well as of the parent compound were calculated using the ACD/Percepta 14.0.0 (ACD/Labs, 2015 Release) software. Then the multivariate chemometric analysis (PCA) was performed in order to compare the toxicity of the photoproducts and the toxicity assessment methods. Data preprocessing and PCA analysis were performed using the R 3.2.3 software (GNU project). The obtained data was centered and scaled before the chemometric analysis.

## 3. Results and Discussion

### 3.1. Optimization of the LC–ESI–MS/MS Method

The chromatographic conditions were optimized based on the UHPLC reversed-phase C18 column and various organic modifiers, buffers, and elution systems including gradient elution were tested. Finally, the gradient elution with a mixture of acetonitrile and water with addition of 0.1% of formic acid as a mobile phase was chosen.

The choice between the two most frequently used in LC–MS ionization methods, electrospray ionization (ESI) and atmospheric pressure chemical ionization (APCI), was made on the basis of the preliminary study and the comparison of these sources for the investigation of phototransformation process of several psychotropic drugs including sertindole. According to this research, ESI outperformed APCI [15].

### 3.2. Quantitative Study of the Phototransformation Process

#### 3.2.1. Calibration and Validation of the Method

The calibration curve was linear over the concentration range (0.5–20 μg mL^−1^), and satisfied the following equation: *y* = 4.5894 (±0.01143)*x* + 0.45228 (±0.1809) (where *y* is sertindole concentration and *x* stands for a peak area) with the correlation coefficient 0.9998. The complete data concerning a quantitative method calibration including inter- and intra-day precision, LOD, and LOQ is presented in Table 1 and Table S1. The obtained equation was then used to calculate sertindole concentration during the photodegradation experiments. Robustness of the method was also tested and the obtained results are presented in Table S2.

#### 3.2.2. Phototransformation Kinetics

**Figure 1:**
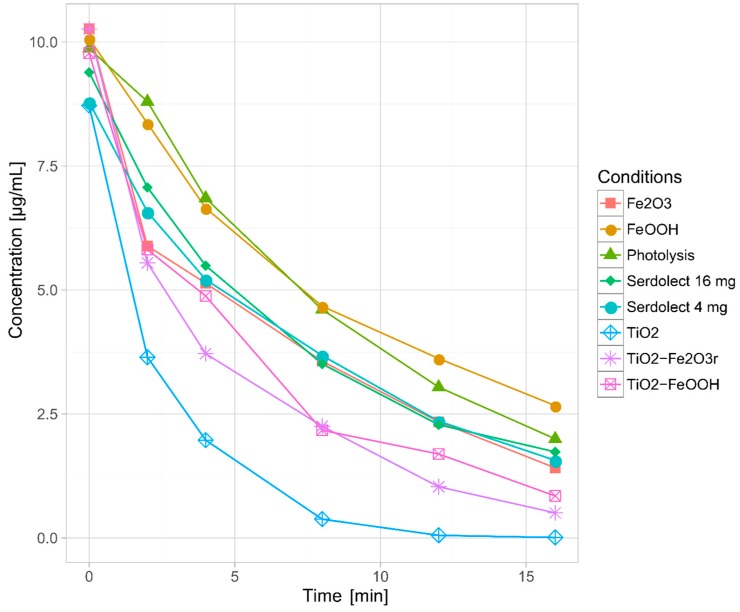
Kinetics of sertindole photodecomposition.

In all the studied cases the photodecomposition reaction fitted the pseudo-first order model (ln c = ln c_0_ – kt). Half-life of sertindole during the direct photolysis process was almost the highest amongst all the conducted experiments and amounted to 6.96 min. Only in the FeOOH-mediated photocatalysis half-life was higher (8.36 min). All the remaining photocatalytic processes as well as experiments with pharmaceutical formulations resulted in faster decomposition of the parent compound (Figure 1). As it was expected, the lowest half-life was observed in the TiO_2_-mediated photocatalysis (1.67 min). The summary of the sertindole photodecomposition kinetics is presented in Table 2.

Taking into account the obtained results, sertindole should be considered as a highly photolabile compound and should be protected from light. Unsurprisingly, the addition of the photocatalysts generally accelerated the degradation of the parent compound. The most spectacular results were achieved by adding TiO_2_, which was expected, as this compound is generally considered to be the most effective amongst the photocatalysts [4]. After 16 min of irradiation, sertindole was almost undetectable. On the other hand, the application of FeOOH resulted in the slowest degradation rate—even slower than in the case of direct photolysis. This phenomenon could be explained by a very low photocatalytic efficiency combined with the shielding effect—the presence of the catalyst increases the turbidity of the sample (which decreases the penetration of radiation), and weak photocatalytic activity is not capable to compensate such an effect. The application of Fe_2_O_3_ noticeably increased the degradation rate in comparison with the direct photolysis and was significantly more active than FeOOH, which is consistent with previous studies [16]. Sertindole half-lives obtained in the experiments with the use of photocatalysts mixtures corresponded with the aforementioned results—the mixture of TiO_2_ and Fe_2_O_3_ catalyzed the photodecomposition more efficiently than TiO_2_–FeOOH mixture. The results of the pharmaceutical formulations experiments were particularly interesting. Half-lives in both cases were slightly lower than in the direct photolysis, but the differences between Serdolect^®^ 4 mg (S4) and Serdolect^®^ 16 mg (S16) were rather insignificant. This finding suggests that in the S4 and S16, photocatalytic processes TiO_2_ played the main role (at least from the quantitative point of view) and the presence of iron oxides did not substantially alter the degradation rate. Additionally, it should be noted that despite the shielding effect caused by photocatalytic-inactive excipients, the decomposition of sertindole was faster than in the direct photolysis. This suggests that photocatalysts present in the pharmaceutical formulations could play a significant role in the phototransformation processes and their influence on the formation of the TPs should be also studied.

In order to examine the influence of hydrolysis or adsorption on the TiO_2_, Fe_2_O_3_, FeOOH particles or on the substances present in the pharmaceutical formulations of S4 and S16, the dark control samples were performed simultaneously. No significant decrease in sertindole concentration was observed in the direct photolysis, photocatalysis, or pharmaceutical formulations experiments, which indicates that both hydrolysis and adsorption did not play any role in the removal of the parent compound.

### 3.3. Identification of the Transformation Products

The qualitative analysis of the phototransformation process generally was done with the use of auto MS/MS mode, automatically selecting the precursor ions for the fragmentation. However, in some cases the acquisition mode was changed to the targeted MS/MS, as well as some MS/MS experiments were repeated with the use of various collision induced dissociation (CID) energy in order to obtain maximum information about the TPs structure. MS/MS spectra of sertindole and its TPs, along with the suggested fragmentation patterns, are shown in the supplementary material (Figure S1–S19).

**Figure 2:**
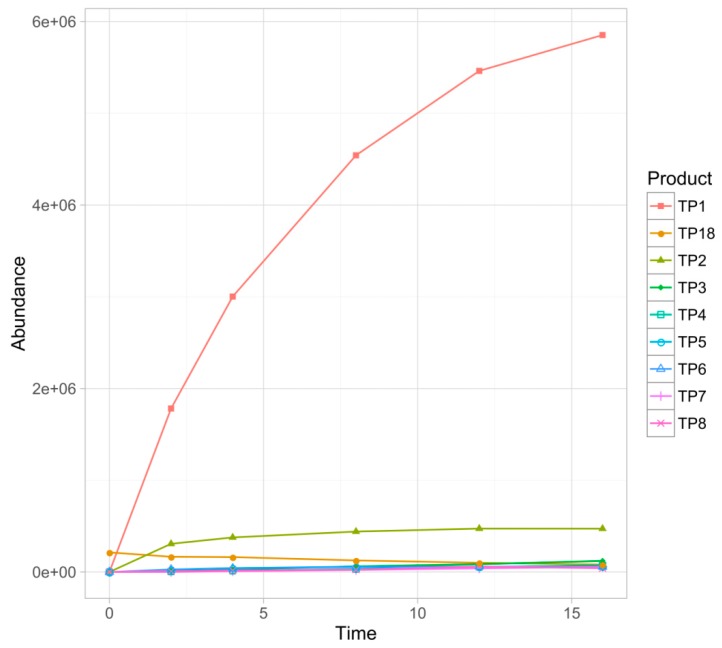
Evolution profiles of the phototransformation products (TPs) formed in the direct photolysis.

**Figure 3:**
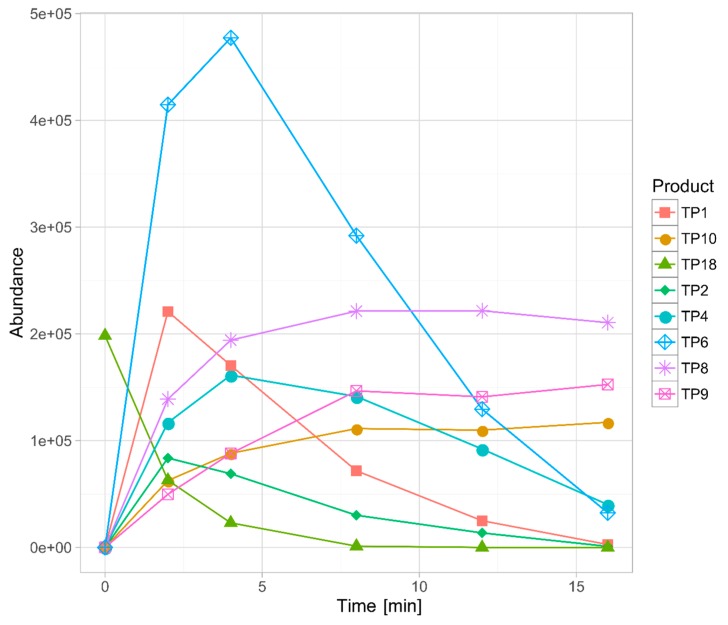
Evolution profiles of the TPs formed in the TiO_2_ photocatalysis.

**Figure 4:**
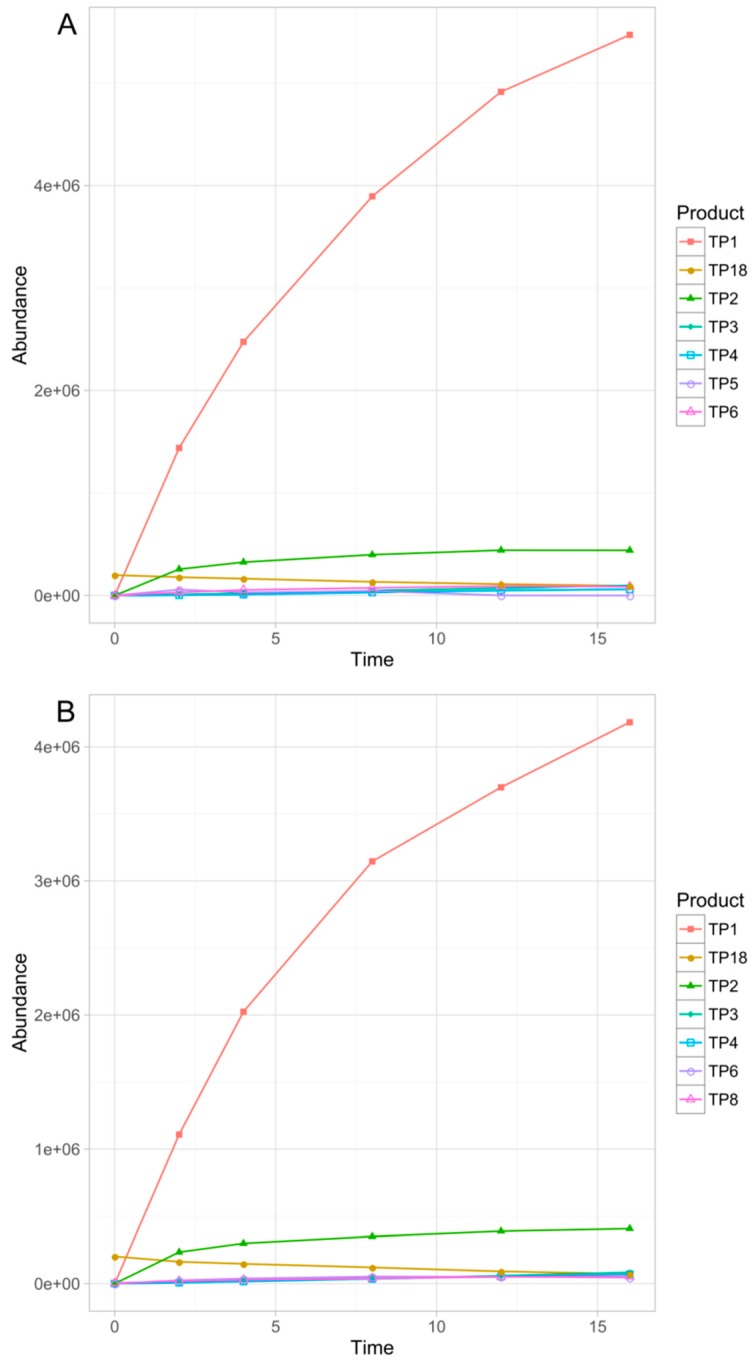
Evolution profiles of the TPs formed in FeOOH (**A**) and Fe_2_O_3_ (**B**) photocatalysis.

**Figure 5:**
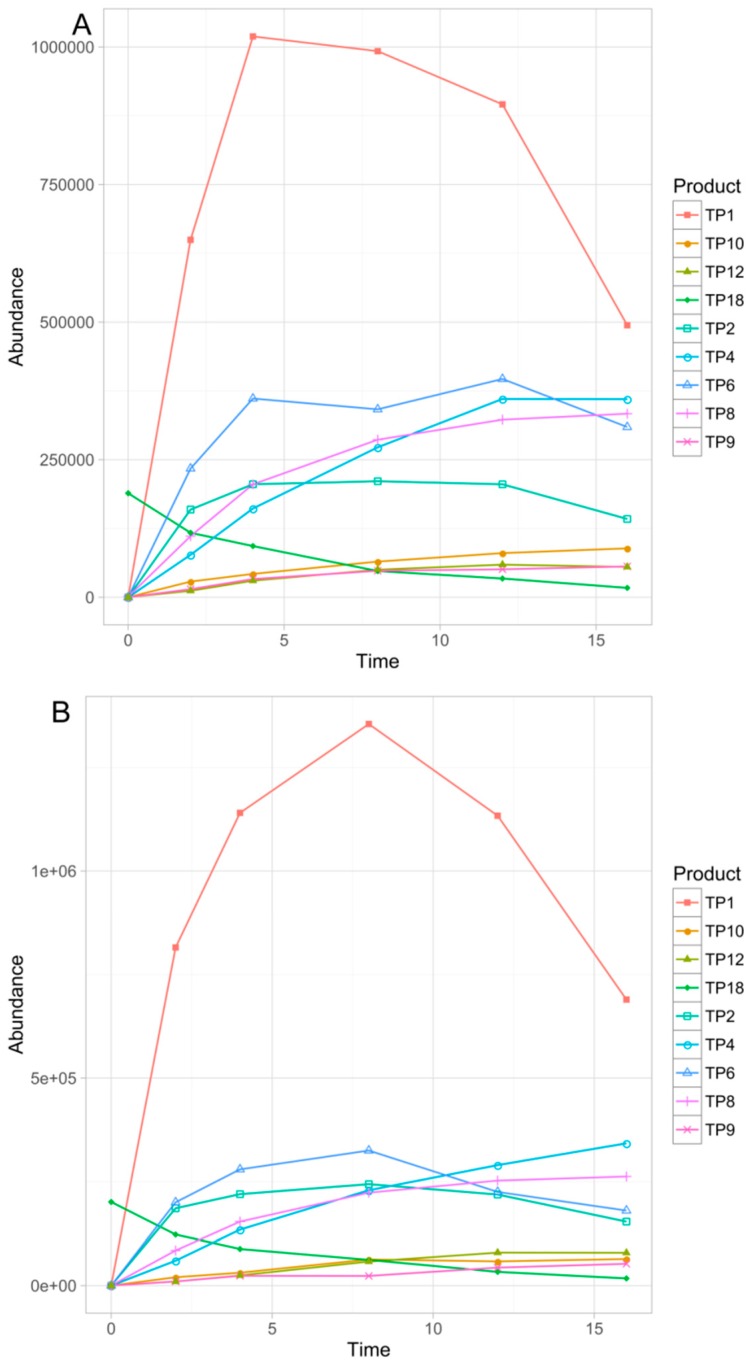
Evolution profiles of the TPs formed in TiO_2_–FeOOH (**A**) and TiO_2_–Fe_2_O_3_ (**B**) photocatalysis.

**Figure 6:**
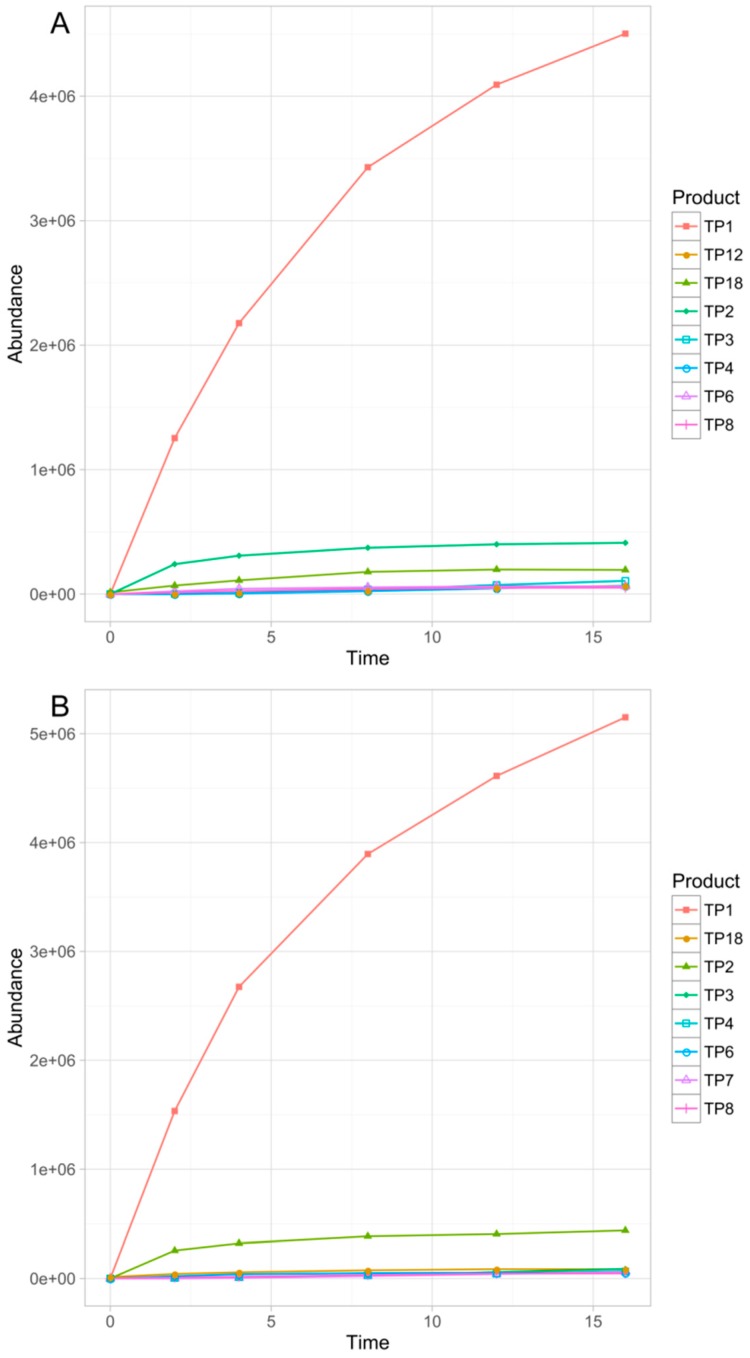
Evolution profiles of the TPs formed in Serdolect^®^ 4 mg (**A**) and Serdolect^®^ 16 mg (**B**) experiments.

Overall, eighteen TPs were formed in the studied phototransformation processes, and their structures were elucidated on the basis of the obtained MS/MS spectra (Table 3). Half of the identified TPs were formed during all the experiments, however, in some cases only at a trace level. The remaining TPs were absent at least in one sample. The evolution profiles of the TPs formed in the experiments are presented in Figure 2, Figure 3, Figure 4, Figure 5 and Figure 6 (TPs detected at a trace level are not shown).

MS/MS spectrum of sertindole (CID energy = 25.1 eV) is shown in Figure S1. The fragmentation began with the elimination of an imidazolidine-2-one and the formation of *m/z* 355.1379 ion, then the elimination of an ethyl fragment (*m/z* 329.1200). Next the gradual decomposition of a piperidine ring took place (*m/z* 298.0765 and *m/z* 270.0464 ions). The main fragmentation ion—*m/z* 113.0713—represented 1-ethenyl-imidazolidine-2-one, fragment common for the majority of TPs with an unchanged imidazolidine-2-one ring. The ions representing various fragments of 1-[2-(piperidine-1-yl)ethyl]imidazolidine-2-one were also present in the spectrum (*m/z* 196.1443, *m/z* 168.1131, and *m/z* 142.0967 ions).

TP1 was the major product in almost all of the experiments. Only in the case of TiO_2_-mediated photocatalysis its abundance did not significantly diverge from the other TPs. In the direct photolysis (its DAD peak area equaled over 290% of sertindole peak area after 16 min of irradiation), Fe_2_O_3_ and FeOOH photocatalysis as well as in the S4 and S16 experiments, the abundance of TP1 was over one order of magnitude higher than any of the remaining TPs. Its concentration was increasing up to 16 min when the experiments were terminated. In the mixed-catalysts experiments, TP1 was also the main product, however, its abundance was less than one order of magnitude higher than the other TPs. Its kinetic behavior was also different. In the TiO_2_–FeOOH experiment TP1 concentration was increasing up to 4 min and then the product started to decompose. In the TiO_2_–Fe_2_O_3_ the photocatalysis decomposition of TP1 started after 8 min. In the TiO_2_-mediated photocatalysis TP1 was the second most abundant product and its kinetic behavior was similar to that observed in TiO_2_–FeOOH experiment. TP1 contained one additional oxygen atom instead of chlorine. Based on its MS/MS spectrum (Figure S2), the whole 1-[2-(piperidine-1-yl)ethyl]imidazolidine-2-one fragment remained unchanged. Therefore, the additional oxygen was attached to the phenylindole fragment. Taking into account that in the case of this product dechlorination took place, TP1 was probably a product of chlorine substitution with a hydroxyl group.

TP2 was the second most abundant product in all the experiments excluding TiO_2_, TiO_2_–FeOOH, and TiO_2_–Fe_2_O_3_ photocatalysis, and was probably a product of double *N*-oxidation of TP1. This assumption was based on a very feasible loss of two additional oxygen atoms (*m/z* 439.2115 and *m/z* 421.1996) without any fragmentation of 1-[2-(piperidine-1-yl)ethyl]imidazolidine-2-one fragment (Figure S3).

TP3 was not detected in the TiO_2_-catalyzed sample, and in TiO_2_–FeOOH as well as in TiO_2_–Fe_2_O_3_ experiments only its traces were detected. In the remaining experiments it was the third most abundant product (except the S4 where TP18 was more abundant). TP3 was probably a product of TP1 defluorination—its fragmentation pattern (Figure S4) was very similar to TP1 but the fragmentation ions did not contain fluorine.

TP4 was formed in all the experiments and its highest abundance was found in the TiO_2_–FeOOH and TiO_2_–Fe_2_O_3_ samples. It was a product of TP1 hydroxylation and dehydrogenation. An additional oxygen was probably attached to a phenylindole fragment based on the presence of 1-[2-(piperidine-1-yl)ethyl]imidazolidine-2-one fragment without any oxygen atom (Figure S5). However, an unusual fragmentation of this ion (*m/z* 168.1123 and *m/z* 142.0950) suggested that dehydrogenation took place in the piperidine ring.

TP5 was found in trace level in the majority of experiments and was completely absent in S16 sample. However, it was the sixth most abundant product in the direct photolysis and FeOOH-catalyzed samples. TP5 was a product of addition of two oxygen atoms to a sertindole molecule. Both of them were eliminated quite easily (*m/z* 439.1637, Figure S6), therefore, they were rather not attached to an aromatic rings. Nevertheless, a significant difference in the behavior of these two oxygen atoms was observed—only one of them was present in the ions containing the residuals of a piperidine ring (such as *m/z* 315.0954 and *m/z* 182.0912). Such observations and the presence of an unchanged 1-ethenyl-imidazolidine-2-one fragment (*m/z* 113.0709 ion) suggested that TP5 was a piperidine *N*-oxide containing a hydroxyl group attached to a piperidine ring. A very similar fragmentation pattern was observed in the case of TP10 (Figure S11) which was probably an isomer of TP5.

TP6 was detected in all the experiments and was the major product in the TiO_2_, TiO_2_–FeOOH, and TiO_2_–Fe_2_O_3_ samples. In this case the hydroxylation of an imidazolidine-2-one ring took place, which was confirmed by the presence of *m/z* 129.0674 (1-ethenyl-imidazolidine-2-one with an additional oxygen) and *m/z* 111.0553 (dehydrogenated 1-ethenyl-imidazolidine-2-one) ions (Figure S7). The presence of *m/z* 355.1355 ion confirmed that hydroxylation did not take place in an ethyl fragment.

TP7, the product of elimination of 4-fluorophenyl from TP4, generally was detected in trace level. In the TiO_2_-catalyzed experiment it was completely absent. Only in cases of the direct photolysis and S16 relatively high abundances were observed. The fragmentation pattern of TP7 (Figure S8) was in general very similar to TP4 but the fragmentation ions did not contain the 4-fluorophenyl fragment. Both additional oxygen atoms were not feasibly eliminated (which suggested aromatic hydroxylation) and a piperidine ring was susceptible to the fragmentation (*m/z* 168.1123 and *m/z* 142.0981 ions).

TP8 was formed during all the conducted experiments, however, in the direct photolysis, FeOOH photocatalysis and S16 experiments it was detected in trace level. The product was formed probably as a consequence of aromatic hydroxylation of sertindole. The ions representing 1-[2-(piperidine-1-yl)ethyl]imidazolidine-2-one and its fragments remained unchanged (*m/z* 142.1247 and *m/z* 113.0713 ions, Figure S9).

TP9, detected in the TiO_2_, TiO_2_–FeOOH, TiO_2_–Fe_2_O_3_ samples and as the traces in the direct photolysis as well as in Fe_2_O_3_ photocatalysis, possessed three additional oxygen atoms. One of them was attached to 1-ethenyl-imidazolidine-2-one fragment (*m/z* 129.0662 and *m/z* 111.0547 ions, Figure S10), the second one to a piperidine ring (*m/z* 210.1219 ion), and the third one was probably an aromatic hydroxyl group.

TP11 contained one additional hydroxyl group instead of chlorine. The fragmentation patterns of TP11 and the parent compound were very similar (Figure S12), therefore, in the case of this product the substitution of fluorine with a hydroxyl group probably took place. TP11 was found in all the experiments, however, only at a trace level.

TP12 was formed probably as a consequence of aromatic hydroxylation of TP1. In its MS/MS spectrum (Figure S13) unmodified 1-[2-(piperidine-1-yl)ethyl]imidazolidine-2-one fragments were observed (*m/z* 194.1247 and *m/z* 113.0707 ions) and the additional hydroxyl groups were not eliminated easily (e.g., *m/z* 310.1222 and *m/z* 327.1488 ions). TP12 was generally detected as the traces and only in the TiO_2_–Fe_2_O_3_, TiO_2_–FeOOH, and S4 experiments it was present in a relatively high level.

TP13 possessed an additional double bond in a piperidine ring (unusually high abundance of *m/z* 168.1122 ion—a consequence of piperidine fragmentation, Figure S14) and three additional oxygen atoms. None of them was feasibly eliminated, therefore, all of them probably formed aromatic hydroxyl groups. TP13 was generally a low-abundant product (in the TiO_2_ photocatalysis the product was not detected).

TP14 was a product of sertindole piperidine ring hydroxylation which was confirmed by an unusually high abundance of 1-[2-(piperidine-1-yl)ethyl]imidazolidine-2-one fragmentation ion (*m/z* 142.0964) and an unchanged 1-ethenyl-imidazolidine-2-one fragment (*m/z* 113.0709). TP14 was not formed during the direct photolysis. In the remaining experiments it was detected at a trace level.

TP15 possessed two additional double bonds which were probably located in a piperidine ring (*m/z* 325.0877 and *m/z* 308.0628 ions), and was a low-abundant product in all the experiments.

TP16 was a product of TP15 hydroxylation. The location of two additional double bonds was confirmed, similarly as in the case of TP15, by the presence of *m/z* 325.0861, *m/z* 308.0643, and additionally *m/z* 351.1076 (Figure S17). A hydroxyl group was attached to an imidazolidine-2-one ring, which was confirmed by the presence of *m/z* 129.0654 and *m/z* 111.0547 ions. TP16 was not detected in the direct photolysis, TiO_2_–Fe_2_O_3_, TiO_2_–FeOOH, and S16 experiments. In the remaining experiments it was detected at a trace level.

The dehydrogenation of TP16 gave TP17 product whose occurrence was very similar. Its fragmentation pattern is shown in Figure S18.

TP18, a dechlorinated sertindole derivative (Figure S19), was a particularly interesting product. In all the experiments, excluding S4 and S16, this species was present from the beginning of the experiments and its concentration was decreasing up to 16 min. Unsurprisingly, the fastest degradation was observed in the TiO_2_ photocatalysis. These observations suggested that TP18 is an impurity, present in the sertindole standard but absent in both pharmaceutical formulations. Noteworthy is that in the S4 and S16 experiments the opposite kinetic behavior of this product was observed—it was almost undetectable at the beginning, and its concentration was increasing up to 16 min. Moreover, TP18 reached a significantly higher concentration in S4 than S16 experiment.

**Figure 7:**
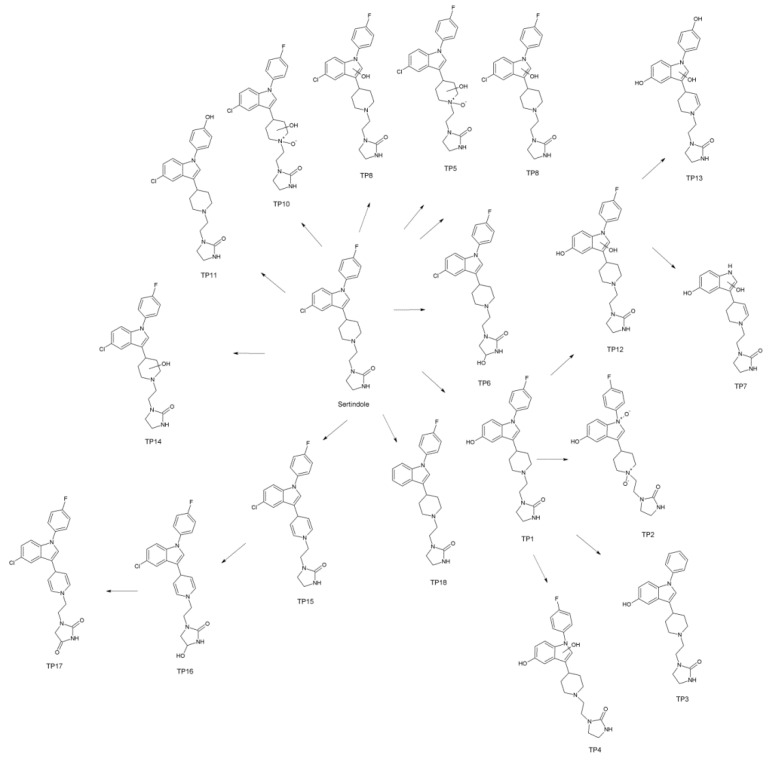
Proposed pathway of sertindole photodegradation.

Proposed photodegradation pathway of sertindole is presented in Figure 7.

### 3.4. Chemometric Data Analysis

In order to perform the multivariate chemometric analysis, all the obtained LC–MS profiles (48 chromatograms, 6 for each phototransformation experiment—Figure S20) recorded in TOF (MS) mode were aligned with MPP software giving 179 entities. After a build-in MPP filtration by flags filtering, abundance (5%–80%) and Kruskal–Wallis test (p = 0.0.1 cut-off) 52 entities were finally selected for the chemometric study.

**Figure 8:**
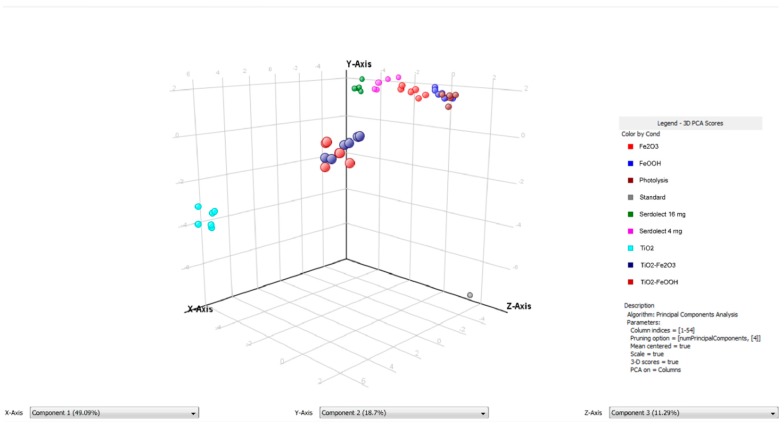
3D principal component analysis (PCA) plot of irradiated and non-irradiated sertindole samples.

The principal component analysis (PCA) based on this data is shown in Figure 8. The PCA is a chemometric technique, widely used for the exploratory data analysis and samples relationships visualization. Its principle relies on the conversion of the original variables to the equal number of latent variables (principal components), which are orthogonal, and explain the largest percent of the data variance. The first three principal components explained 79.08% of the total variance. The distribution of the samples (spheres of each color represent each experiment) shows that TiO_2_-catalyzed samples (turquoise spheres) were the most distant (and therefore the least similar) from the standard samples (sertindole working solution before the experiment, gray spheres). Mixed catalysts samples were placed between these extreme conditions. No significant differences between TiO_2_–Fe_2_O_3_ (dark red spheres) and TiO_2_–FeOOH (dark blue spheres) were observed, however, TiO_2_–Fe_2_O_3_ seem to be a little closer to the remaining samples. The direct photolysis (brown spheres), Fe_2_O_3_ (red spheres), FeOOH (blue spheres) photocatalysis, and pharmaceutical formulations (pink spheres for S4 and green spheres for S16) samples were distributed along the *Z*-axis. As it can be seen, FeOOH samples were the most similar to the direct photolysis which should be explained by its low catalytic activity. Nevertheless, despite the sertindole decomposition in FeOOH-catalyzed experiment was slower than during the direct photolysis, in the TPs profile the effects of photocatalysis are pronounced. Such an observation indicates that the presence of this compound as a pigment could affect the formation of the transformation products. Fe_2_O_3_ samples formed a more outlying group which could be explained by a higher activity of this catalyst. The relationships between FeOOH and Fe_2_O_3_ resemble the relationships between S4 and S16 pharmaceutical formulations—which reflects the influence of the applied pigments on the studied process. Generally, it should be noted that the presence of FeOOH and Fe_2_O_3_ as pigments could affect (however not to a large extent) the phototransformation processes and it should be taken into account during the analysis of the pharmaceutical formulations.

### 3.5. In Silico Evaluation of TPs Properties

In this study the acute toxicity to rodents and mutagenicity of sertindole TPs as well as the parent compound are studied. Additionally, since sertindole is a well-known potassium channel blocker, a probability of hERG inhibition was also calculated. In the case of toxicity to rodents, where six models were applied, PCA was performed. This multivariate exploratory chemometric technique enables the reduction of dimensionality of the data matrices and visualization of relationships between compounds as well as between models.

#### 3.5.1. Acute Toxicity to Rodents

**Figure 9:**
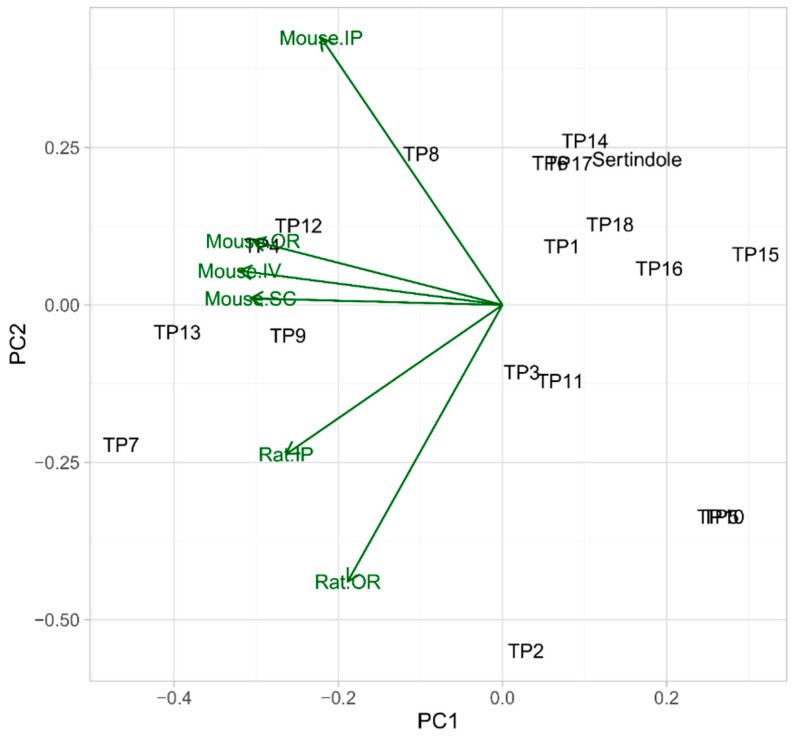
Comparison of acute toxicity to rodents of sertindole and its TPs by PCA.

In silico toxicity to rodents of sertindole as well as its TPs were assessed with the use of ACD/Percepta 14.0.0 (ACD/Labs, 2015 Release). Six toxicity categories were applied: two for rats—oral (OR) and intraperitoneal (IP) and four for mice—oral (OR), intravenous (IV), subcutaneous (SC), intraperitoneal (IP). The calculated LD_50_ values (expressed in log_mg kg_^−1^) are presented in Table S3. In order to visualize the relationships between the toxicity of the analyzed compounds, a principal component analysis was performed. The first principal component (PC1) explained 65.85% of the total variability and the second principal component (PC2)—19.78%. As shown in Figure 9 (toxicity decreases in parallel with increasing LD_50_ values), TP1, TP6, TP14–TP18, and sertindole possessed very similar toxicity to rodents. TP4, TP8, TP9, TP12, and TP13 were generally less toxic for all the models (in the case of TP8—with the exception of the rat oral model which predicted almost the same toxicity). TP3, TP11, and especially TP2 were less toxic for the rat models and possessed very similar toxicity to mice. TP7 should be considered as the least toxic amongst the studied compounds, especially to rats. TP5 and TP10 were more toxic to mice than the parent compound, however, on the other hand, their toxicity to rats was significantly lower.

#### 3.5.2. Mutagenicity

The mutagenicity of sertindole and its TPs expressed as the probability of a positive result of a commonly applied Ames test is shown in Table 4. The majority of the TPs possessed higher predicted mutagenicity, however, only in the case of TP2 and TP15–TP17 the probability exceeded 0.5. Such findings suggest that the introduction of two double bonds into the piperidine ring of the sertindole molecule significantly increases the mutagenicity of the compound. Moreover, as TP2 belongs to the major photodegradation products, the interaction of sertindole with UV-Vis radiation could result in the higher mutagenic potential.

#### 3.5.3. hERG Inhibition

Sertindole is a well-known blocker of hERG potassium channel, which could result in the prolongation of QT interval on the electrocardiogram [9]. Therefore, hERG inhibitory potential of the sertindole TPs was also evaluated. The applied model has correctly predicted the parent compound properties—the calculated probability of inhibition (K_i_ < 10 μM) was 0.95, which was the highest value among the studied compounds. Very similar values were obtained for TP8 and TP18 which suggest that neither dechlorination nor hydroxylation of an indole moiety leads to the decrease of hERG inhibitory potential. On the other hand, double N-oxidation (TP2), the removal of a 5-fluorophenyl moiety (TP9) or dehydrogenation of a piperidine ring (TP4, TP13 and TP15–TP17) significantly reduces the probability of hERG inhibition.

## 4. Conclusions

In this study the photostability of sertindole under the simulated solar radiation was evaluated. On the contrary to the available literature, the studied compound turned out to be highly photolabile—after 16 min of irradiation more than 75% of the initial amount was decomposed. Amongst the formed TPs the major one was a product of sertindole dechlorination followed by hydroxylation. The remaining TPs—the products of aromatic and aliphatic hydroxylation, oxidation, *N*-oxidation, dehydrogenation, and dehalogenation—were significantly less abundant.

Additionally, the influence of three metal oxides, commonly used as pigments in pharmaceutical formulations, on the qualitative and quantitative aspect of the phototransformation process was studied. TiO_2_ influenced the process to the largest extent and after 16 min sertindole was practically undetectable. The impact of two iron oxides, FeOOH and Fe_2_O_3_ was significantly lower—FeOOH slightly inhibited the degradation while Fe_2_O_3_ accelerated the process. The application of the mixtures containing TiO_2_ and iron oxides gave the analogous results—in both cases an increase in the degradation rate was observed, however, in the case of TiO_2_–FeOOH mixture to a less extent. The obtained results were compared with the experiments using the real pharmaceutical formulations—Serdolect^®^ 4 mg and Serdolect^®^ 16 mg, containing TiO_2_ with FeOOH and TiO_2_ with Fe_2_O_3_, respectively, as the excipients. The degradation rates were very similar in both cases and higher than in the direct photolysis, which should be attributed to the activity of TiO_2_. The exploration of the results with use of PCA revealed close relationship between the profiles obtained from FeOOH, Fe_2_O_3_, and the pharmaceutical formulations samples, which indicates that the application of iron oxides as pigments could affect both a quantitative and qualitative aspect of the phototransformation process. From this point of view the other metal oxides characterized by lower photocatalytic activity should be considered as pigments used in pharmaceutical formulations, especially in the case of titanium oxide.

In silico assessment of toxicity showed that the majority of the TPs are less toxic to rodents than the parent compound. On the other hand, several products, including relatively abundant TP2, could possess a significant mutagenic potential. Most of the TPs were marked by high hERG inhibitory potential, however, none of the products surpassed sertindole in this regard.

## Figures and Tables

**Table 1:** Validation results of the developed method with diode array detection (DAD) detection.

Parameters	Results
Linearity	
Concentration range (μg mL^−1^)	(0.5:20)
Slope	4.5894
SD^a^ of slope	0.1143
Intercept	0.4528
SD^a^ of intercept	0.1809
Correlation coefficient *(r*)	0.9998
LOD^b^ (μg mL^−1^)	0.034
LOQ^c^ (μg mL^−1^)	0.100
Precision (RSD^d^%)	
Intra-day (*n* = 12)	1.74
Inter-day (*n* = 18)	2.53
Accuracy	
Recovery (%)	100.20
RSD^d^ (%)	2.77

^a^ standard deviation; ^b^ limit of detection; ^c^ limit of quantification; ^d^ relative standard deviation.

**Table 2:** Summary of sertindole degradation kinetics parameters.

Experiment	Model	Fit (*r*)	*k ± SD* (min^−1^)	*t_1/2_* (min)
Direct photolysis	Pseudo-first-order	0.9989	0.0996 ± 5.22 × 10^−6^	6.96
TiO_2_	Pseudo-first-order	0.9989	0.4143 ± 3.42 × 10^−3^	1.67
FeOOH yellow	Pseudo-first-order	0.9963	0.0829 ± 8.13 × 10^−4^	8.36
Fe_2_O_3_ red	Pseudo-first-order	0.9874	0.1236 ± 8.96 × 10^−4^	5.61
TiO_2_–FeOOH (yellow)	Pseudo-first-order	0.9894	0.1524 ± 9.64 × 10^−5^	4.55
TiO_2_–Fe_2_O_3_ (red)	Pseudo-first-order	0.9950	0.1871 ± 8.61 × 10^−4^	3.70
Serdolect 4 mg	Pseudo-first-order	0.9984	0.1076 ± 2.60 × 10^−3^	6.44
Serdolect 16 mg	Pseudo-first-order	0.9948	0.1052 ± 1.61 × 10^−3^	6.59

**Table 3:** Accurate masses of sertindole phototransformation products.

Cpd.	*t_R_* (min)	Elemental	Mass (*m/z*)		Error (ppm)	DBE	Fragmentation (MS/MS)		Occurrence							
		Formula (M+H)^+^	Exp.	Theo.			Mass (*m/z*)	Elemental Formula	DP^a^	T^b^	Fy^c^	Fr^d^	TFy^e^	TFr^f^	S4^g^	S16^h^
Sert	7.06	C_24_H_27_ClFN_4_O	441.1858	441.1852	1.36	13	355.1379	C_21_H_21_ClFN_2_	+	+	+	+	+	+	+	+
							329.1200	C_19_H_19_ClFN_2_								
							298.0765	C_18_H_14_ClFN								
							270.0464	C_16_H_10_ClFN								
							196.1443	C_10_H_18_N_3_O								
							168.1131	C_8_H_14_N_3_O								
							142.0976	C_6_H_12_N_3_O								
							113.0713	C_5_H_9_N_2_O								
							99.0562	C_4_H_7_N_2_O								
							70.0655	C_4_H_8_N								
TP1	5.50	C_24_H_28_FN_4_O_2_	423.2194	423.2191	0.71	13	337.1704	C_21_H_22_FN_2_O	+	+	+	+	+	+	+	+
							311.1584	C_19_H_20_FN_2_O								
							266.0951	C_17_H_13_FNO								
							196.1443	C_10_H_18_N_3_O								
							168.1123	C_8_H_14_N_3_O								
							142.0976	C_6_H_12_N_3_O								
							113.0713	C_5_H_9_N_2_O								
							84.0810	C_5_H_10_N								
							70.0653	C_4_H_8_N								
TP2	5.48	C_24_H_28_FN_4_O_4_	455.2086	455.2089	−0.66	13	439.2115	C_24_H_28_FN_4_O_3_	+	+	+	+	+	+	+	+
							421.1996	C_24_H_26_FN_4_O_2_								
							194.1250	C_10_H_16_N_3_O								
							113.0712	C_5_H_9_N_2_O								
TP3	5.31	C_24_H_29_N_4_O_2_	405.2283	405.2285	−0.49	13	319.1767	C_21_H_23_N_2_O	+	−	+	+	+ *	+ *	+	+
							196.1445	C_10_H_18_N_3_O								
							113.0712	C_5_H_9_N_2_O								
							84.0807	C_5_H_10_N								
							71.0622	C_3_H_7_N_2_								
TP4	4.72	C_24_H_26_FN_4_O_3_	437.1983	437.1983	0	14	339.1500	C_20_H_20_FN_2_O	+	+	+	+	+	+	+	+
							296.1094	C_18_H_15_FNO_2_								
							194.1286	C_10_H_16_N_3_O								
							168.1123	C_8_H_14_N_3_O								
							142.0950	C_6_H_12_N_3_O								
							113.0705	C_5_H_9_N_2_O								
TP5	5.40	C_24_H_27_ClFN_4_O_3_	473.1742	473.1750	−1.69	13	445.1759	C_23_H_27_ClFN_4_O_2_	+	+ *	+	+ *	+ *	+ *	−	+ *
							439.1637	C_24_H_25_ClFN_4_O								
							387.1273	C_21_H_21_ClFN_2_O_2_								
							359.1309	C_20_H_21_ClFN_2_O								
							316.0954	C_18_H_16_ClFNO								
							194.1297	C_10_H_16_N_3_O								
							182.0912	C_8_H_12_N_3_O_2_								
							113.0709	C_5_H_9_N_2_O								
							85.0768	C_4_H_9_N_2_								
							71.0616	C_3_H_7_N_2_								
TP6	6.89	C_24_H_27_ClFN_4_O_2_	457.1805	457.1801	0.87	13	439.1635	C_24_H_25_ClFN_4_O	+	+	+	+	+	+	+	+
							355.1355	C_21_H_21_ClFN_2_								
							194.1282	C_10_H_16_N_3_O								
							168.1125	C_8_H_14_N_3_O								
							129.0674	C_5_H_9_N_2_O_2_								
							113.0709	C_5_H_9_N_2_O								
							111.0553	C_5_H_7_N_2_O								
							84.0784	C_5_H_10_N								
TP7	2.42	C_18_H_23_N_4_O_3_	343.1778	343.1765	4.08	10	257.131	C_15_H_17_N_2_O_2_	+	−	+ *	+ *	+ *	+ *	+ *	+
							245.1286	C_14_H_17_N_2_O_2_								
							214.0869	C_13_H_12_NO_2_								
							201.0787	C_12_H_11_NO_2_								
							194.1282	C_10_H_16_N_3_O								
							168.1123	C_8_H_14_N_3_O								
							142.0981	C_6_H_12_N_3_O								
							113.0705	C_5_H_9_N_2_O								
TP8	5.93	C_24_H_27_ClFN_4_O_2_	457.1810	457.1801	1.97	13	371.1311	C_21_H_21_ClFN_2_O	+ *	+	+ *	+	+	+	+	+ *
							345.1139	C_19_H_19_ClFN_2_O								
							328.0858	C_19_H_16_ClFNO								
							194.1247	C_10_H_16_N_3_O								
							113.0713	C_5_H_9_N_2_O								
							70.0657	C_4_H_8_N								
TP9	5.28	C_24_H_27_ClFN_4_O_4_	489.1704	489.1699	1.02	13	471.1599	C_24_H_25_ClFN_4_O_3_	+ *	+	−	+ *	+	+	−	−
							443.1607	C_23_H_25_ClFN_4_O_2_								
							387.1255	C_21_H_21_ClFN_4_O_2_								
							361.1126	C_19_H_19_ClFN_2_O_2_								
							210.1219	C_10_H_16_N_3_O_2_								
							129.0662	C_5_H_9_N_2_O_2_								
							113.0707	C_5_H_9_N_2_O								
							111.0547	C_5_H_7_N_2_O								
							69.0505	C_3_H_5_N_2_								
TP10	5.09	C_24_H_27_ClFN_4_O_3_	473.1735	473.1750	−3.17	13	455.1604	C_23_H_27_ClFN_4_O_2_	+ *	+	+ *	+ *	+	+	+ *	+ *
							387.1265	C_21_H_21_ClFN_2_O_2_								
							326.0723	C_19_H_14_ClFNO								
							300.0601	C_17_H_12_ClFNO								
							210.1259	C_10_H_16_N_3_O_2_								
							194.1274	C_10_H_16_N_3_O								
							142.0974	C_6_H_12_N_3_O								
							113.0708	C_5_H_9_N_2_O								
TP11	6.03	C_24_H_28_ClN_4_O_2_	439.1891	439.1895	−0.91	13	353.1431	C_21_H_22_ClN_2_O	+ *	+ *	+ *	+ *	+ *	+ *	+ *	+ *
							196.1421	C_10_H_18_N_3_O								
							113.0712	C_5_H_9_N_2_O								
TP12	4.54	C_24_H_28_FN_4_O_3_	439.2139	439.2140	−0.23	13	353.1632	C_21_H_22_FN_2_O_2_	+ *	+ *	+ *	+ *	+	+	+	+ *
							327.1488	C_19_H_20_FN_2_O_2_								
							310.1222	C_19_H_17_FNO_2_								
							194.1247	C_10_H_16_N_3_O								
							113.0707	C_5_H_9_N_2_O								
TP13	3.89	C_24_H_27_N_4_O_4_	435.2036	435.2027	2.07	14	194.1297	C_10_H_16_N_3_O	+ *	−	+ *	+ *	+ *	+ *	+ *	+ *
							168.1122	C_8_H_14_N_3_O								
							113.0705	C_5_H_9_N_2_O								
TP14	6.35	C_24_H_27_ClFN_4_O_2_	457.1802	457.1801	0.22	13	142.0964	C_6_H_12_N_3_O	−	+ *	+ *	+ *	+ *	+ *	+ *	+ *
							113.0709	C_5_H_9_N_2_O								
TP15	6.57	C_24_H_23_ClFN_4_O	437.1516	437.1539	−5.26	15	325.0877	C_19_H_15_ClFN_2_	+ *	+ *	+ *	+ *	+ *	+ *	+ *	+ *
							308.0628	C_19_H_12_ClFN								
							113.0710	C_5_H_9_N_2_O								
TP16	6.35	C_24_H_23_ClFN_4_O_2_	453.1474	453.1488	−3.09	15	435.1362	C_24_H_21_ClFN_4_O	−	+ *	−	−	+ *	+ *	+ *	−
							351.1076	C_21_H_17_ClFN_2_								
							325.0861	C_19_H_15_ClFN_2_								
							308.0643	C_19_H_12_ClFN								
							129.0654	C_5_H_9_N_2_O_2_								
							113.0707	C_5_H_9_N_2_O								
							111.0547	C_5_H_7_N_2_O								
TP17	6.66	C_24_H_21_ClFN_4_O_2_	451.1304	451.1332	−6.21	16	325.0805	C_19_H_15_ClFN_2_	+ *	+ *	−	−	+ *	+ *	+ *	−
							308.0537	C_19_H_12_ClFN								
							258.0515	C_15_H_10_ClFN								
							127.0502	C_5_H_7_N_2_O_2_								
							99.0568	C_4_H_7_N_2_O								
TP18	6.52	C_24_H_28_FN_4_O	407.2229	407.2242	−3.19	13	321.1769	C_21_H_22_FN_2_	+	+	+	+	+	+	+	+
							196.1423	C_10_H_18_N_3_O								
							113.0710	C_5_H_9_N_2_O								

^*^ Detected in trace level. ^a^ Direct photolysis; ^b^ TiO_2_; ^c^ FeOOH (yellow); ^d^ Fe_2_O_3_ (red); ^e^ TiO_2_–Fe_2_O_3_ yellow; ^f^ TiO_2_–Fe_2_O_3_ red; ^g^ Serdolect 4 mg; ^h^ Serdolect 16 mg.

**Table 4:** Mutagenicity and hERG inhibitory potential of sertindole and its TPs.

Compound	Mutagenicity	hERG inhibition
Sertindole	0.39	0.95
TP1	0.4	0.71
TP2	0.58	0.33
TP3	0.4	0.57
TP4	0.4	0.37
TP5	0.48	0.79
TP6	0.38	0.87
TP7	0.41	0.06
TP8	0.26	0.92
TP9	0.25	0.68
TP10	0.48	0.79
TP11	0.26	0.75
TP12	0.26	0.72
TP13	0.36	0.21
TP14	0.37	0.88
TP15	0.78	0.53
TP16	0.84	0.37
TP17	0.7	0.4
TP18	0.45	0.92

## Supplementary Materials

The following are available online at https://www.mdpi.com/1999-4923/11/7/299/s1, Figure S1: MS/MS spectrum and fragmentation pattern of sertindole, Figure S2: MS/MS spectrum and fragmentation pattern of TP1, Figure S3: MS/MS spectrum and fragmentation pattern of TP2, Figure S4: MS/MS spectrum and fragmentation pattern of TP3, Figure S5: MS/MS spectrum and fragmentation pattern of TP4, Figure S6: MS/MS spectrum and fragmentation pattern of TP5, Figure S7: MS/MS spectrum and fragmentation pattern of TP6, Figure S8: MS/MS spectrum and fragmentation pattern of TP7, Figure S9: MS/MS spectrum and fragmentation pattern of TP8, Figure S10: MS/MS spectrum and fragmentation pattern of TP9, Figure S11: MS/MS spectrum and fragmentation pattern of TP10, Figure S12: MS/MS spectrum and fragmentation pattern of TP11, Figure S13: MS/MS spectrum and fragmentation pattern of TP12, Figure S14: MS/MS spectrum and fragmentation pattern of TP13, Figure S15: MS/MS spectrum and fragmentation pattern of TP14, Figure S16: MS/MS spectrum and fragmentation pattern of TP15, Figure S17: MS/MS spectrum and fragmentation pattern of TP16, Figure S18:, Figure S19: MS/MS spectrum and fragmentation pattern of TP18, Figure S20: Total ion chromatograms (overlay) of irradiated sertindole samples, Table S1: Calibration of the UHPLC method with DAD detection for determination of sertindole, Table S2: Robustness of the UHPLC method with DAD detection for determination of sertindole, Table S3: Toxicity of sertindole and its TPs to rodents.
