# Supplementary material for: Photodegradation Study of Sertindole by UHPLC-ESI-Q-TOF and Influence of Some Metal Oxide Excipients on the Degradation Process
Source: Pharmaceutics. 2019 Jun 27;11(7):299. doi: 10.3390/pharmaceutics11070299 (PMC6680419; doi:10.3390/pharmaceutics11070299)
Supplement: Supplementary file 1 [file pharmaceutics-11-00299-s001.pdf]

pharmaceutics

Supplementary Material: Photodegradation Study of Sertindole by UHPLC-ESI-Q-TOF and Influence of Some Metal Oxide Excipients on the Degradation Process

Jakub Trawiński and Robert Skibiński

Table S1. Calibration of the UHPLC method with DAD detection for determination of sertindole.

Concentration (μg mL^{-1})	Mean peak area (*n* = 3)	RSD (%)
0.5	3.00	4.94
1.0	5.41	2.87
5.0	23.58	1.47
10.0	45.50	0.49
15.0	69.75	0.30
20.0	91.16	2.17

Table S2. Robustness of the UHPLC method with DAD detection for determination of sertindole.

Conditions	Modification	Mean peak area (*n* = 3)	RSD (%)
	5–55	47.26	0.97
Mobile phase composition (A%)	5–60	46.57	1.36
	5–65	47.25	1.54
	0.28	47.36	1.93
Flow rate (ml/min)	0.30	47.40	0.58
	0.32	47.19	0.52
	32	47.83	1.07
Column temp. (ºC)	35	47.29	1.96
	38	47.46	1.17
	258	47.28	1.39
Wavelength (nm)	260	47.29	1.96
	262	46.80	1.99

Table S3. Toxicity of sertindole and its TPs to rodents (LD_{50} values expressed in $\log_{mg\ kg^{-1}}$).

Compound	Mouse IP	Mouse OR	Mouse IV	Mouse SC	Rat IP	Rat OR
Sertindole	2.78	2.76	1.55	2.22	2.31	2.3
TP1	2.76	2.8	1.63	2.31	2.37	2.49
TP2	2.41	2.63	1.67	2.51	2.58	2.94
TP3	2.68	2.89	1.62	2.31	2.49	2.67
TP4	2.99	2.99	1.86	2.74	2.69	2.72
TP5	2.47	2.47	1.47	2.18	2.41	2.68
TP6	2.81	2.83	1.68	2.29	2.41	2.34

TP7	2.85	3.36	1.99	2.65	2.95	3.03
TP8	2.92	2.83	1.74	2.65	2.48	2.44
TP9	2.73	3	1.96	2.75	2.71	2.63
TP10	2.47	2.47	1.47	2.18	2.41	2.68
TP11	2.63	2.87	1.6	2.24	2.49	2.62
TP12	2.95	2.92	1.88	2.78	2.53	2.68
TP13	3.03	3.13	1.94	2.68	2.71	3.02
TP14	2.81	2.78	1.65	2.28	2.34	2.3
TP15	2.69	2.6	1.44	2.15	1.91	2.55
TP16	2.74	2.66	1.57	2.21	2.05	2.62
TP17	2.78	2.77	1.78	2.38	1.94	2.52
TP18	2.73	2.78	1.55	2.29	2.37	2.38

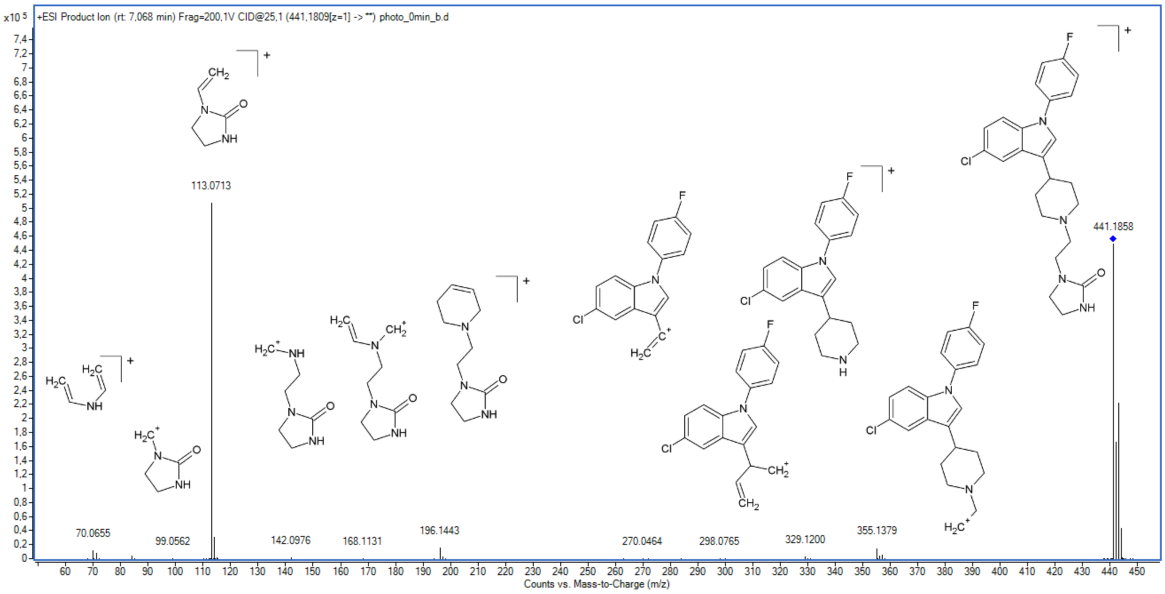

Figure S1. MS/MS spectrum and fragmentation pattern of sertindole.

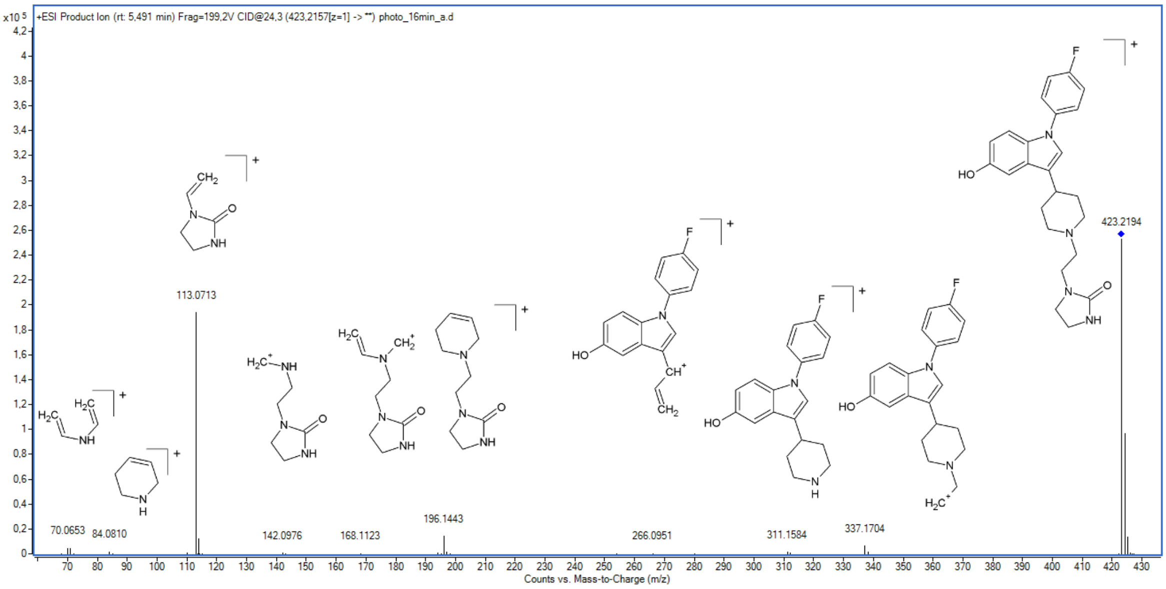

Figure S2. MS/MS spectrum and fragmentation pattern of TP1.

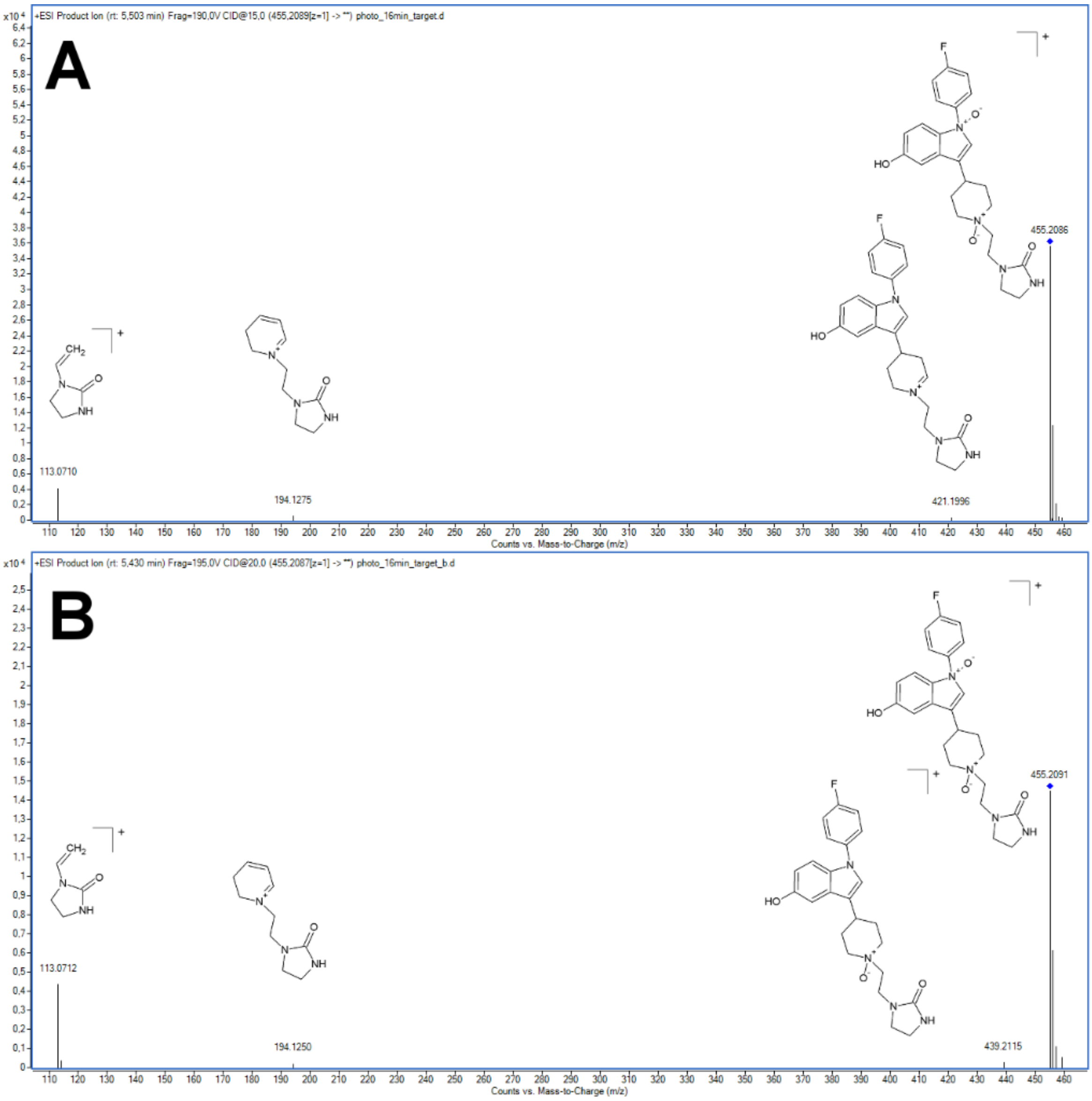

Figure S3. MS/MS spectrum and fragmentation pattern of TP2.

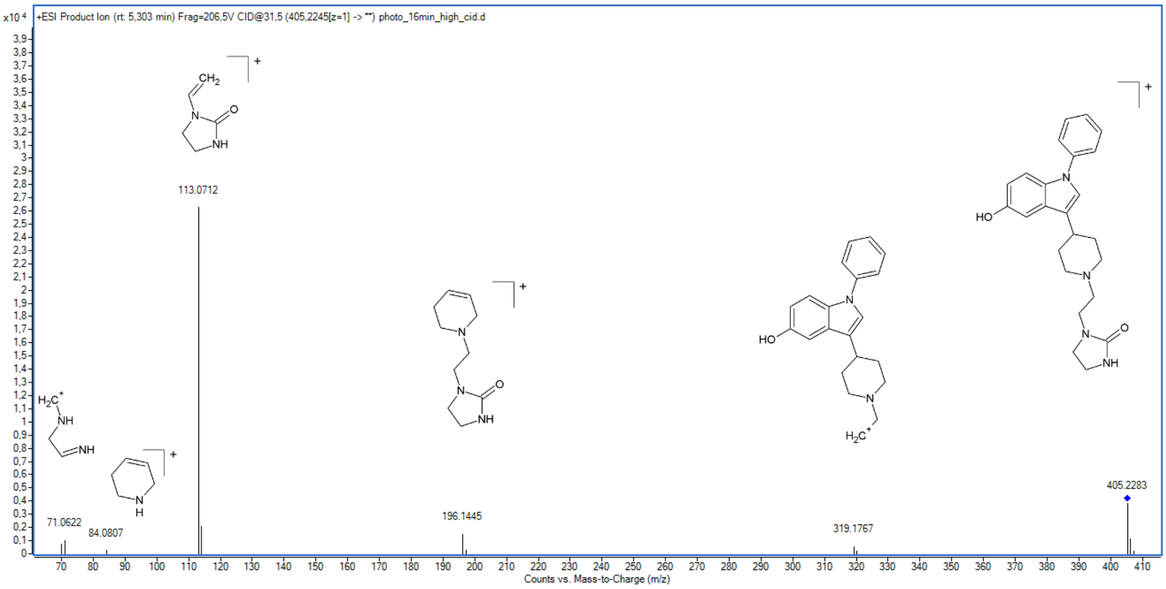

Figure S4. MS/MS spectrum and fragmentation pattern of TP3.

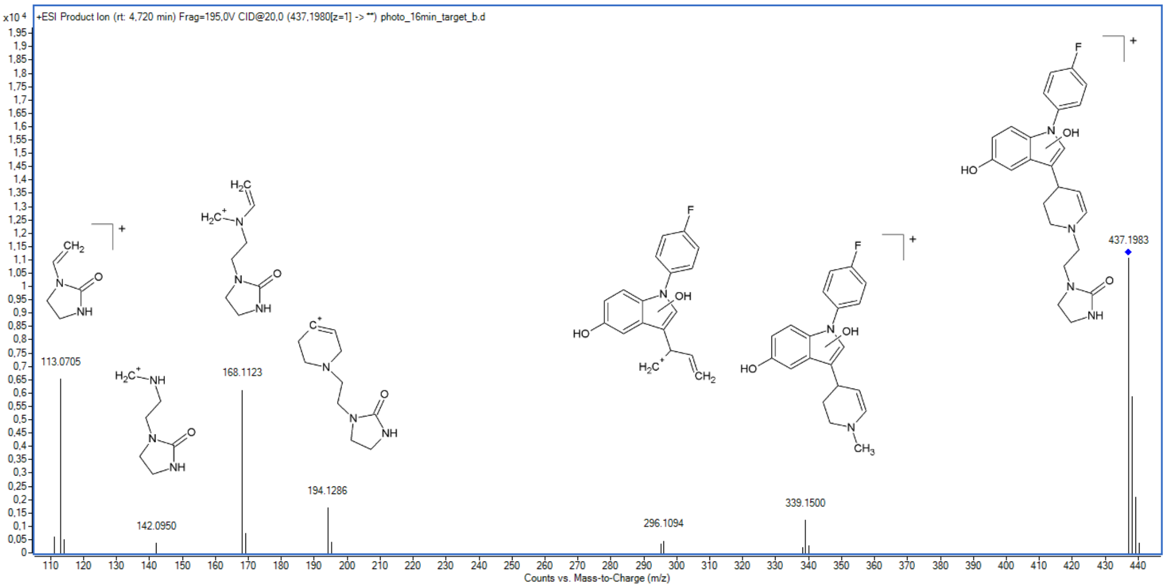

Figure S5. MS/MS spectrum and fragmentation pattern of TP4.

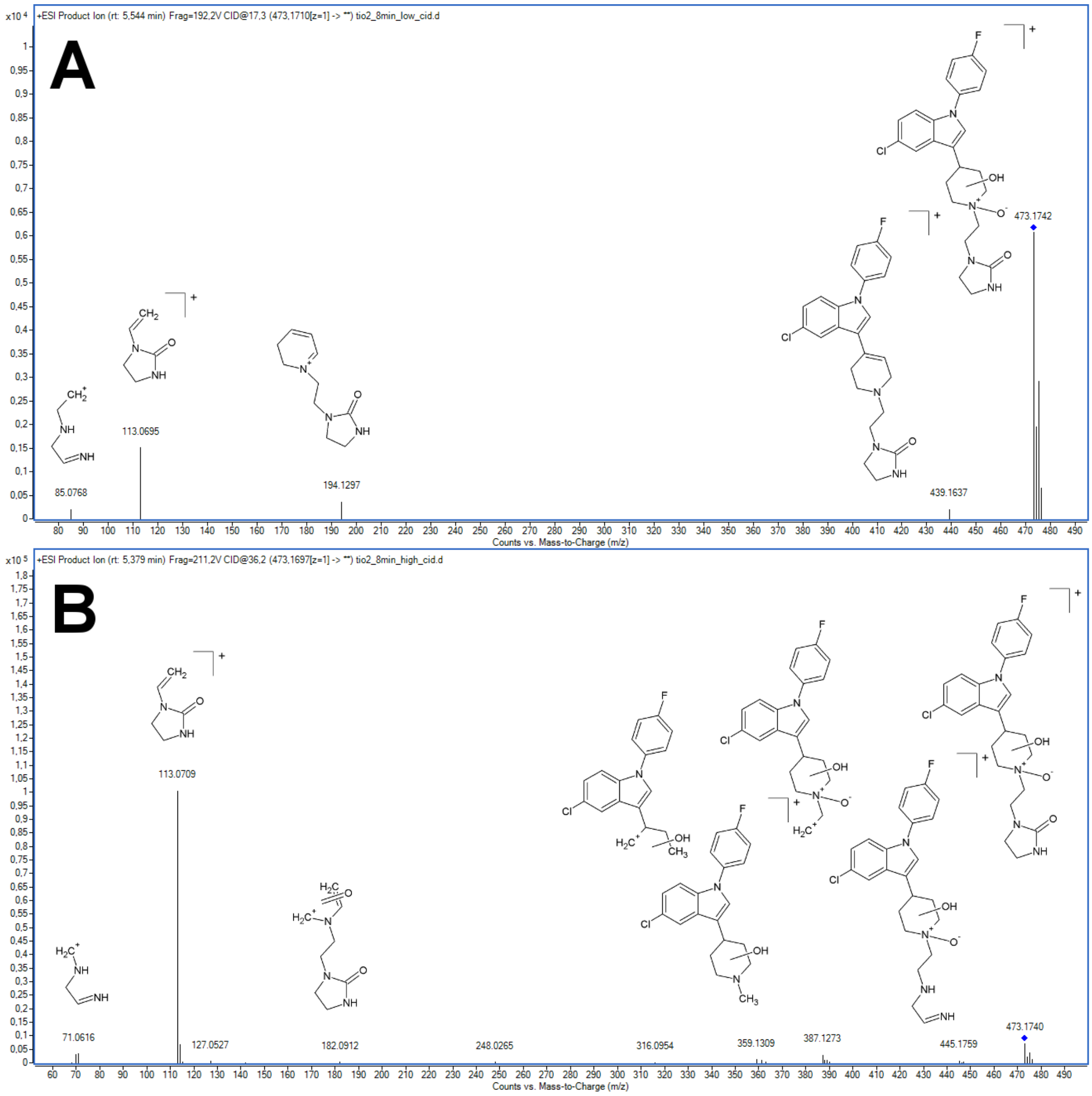

Figure S6. MS/MS spectrum and fragmentation pattern of TP5.

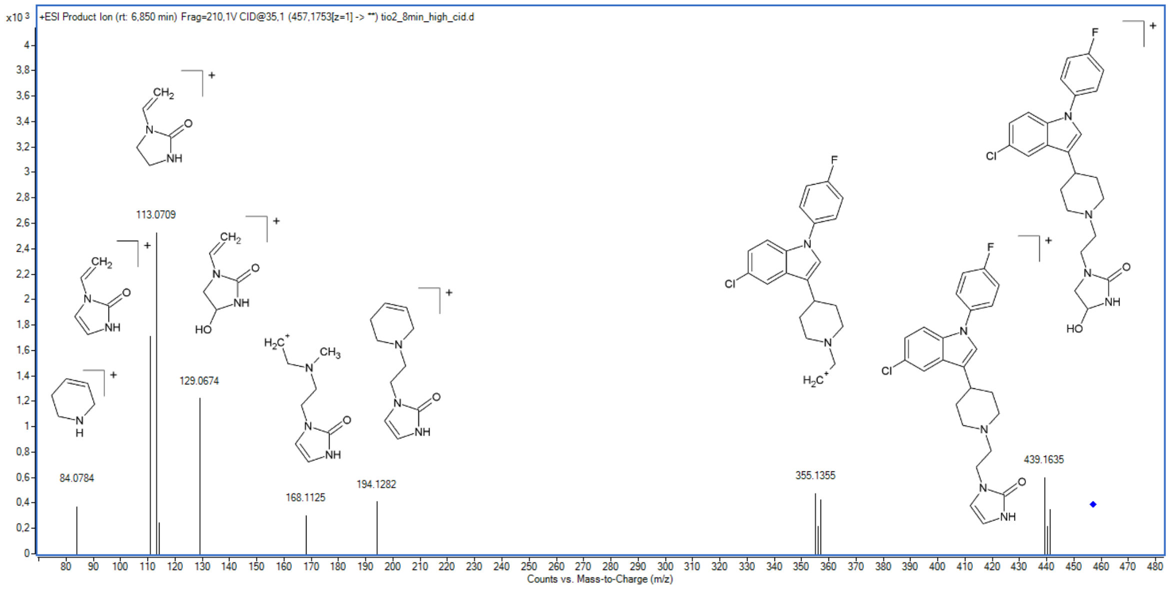

Figure S7. MS/MS spectrum and fragmentation pattern of TP6.

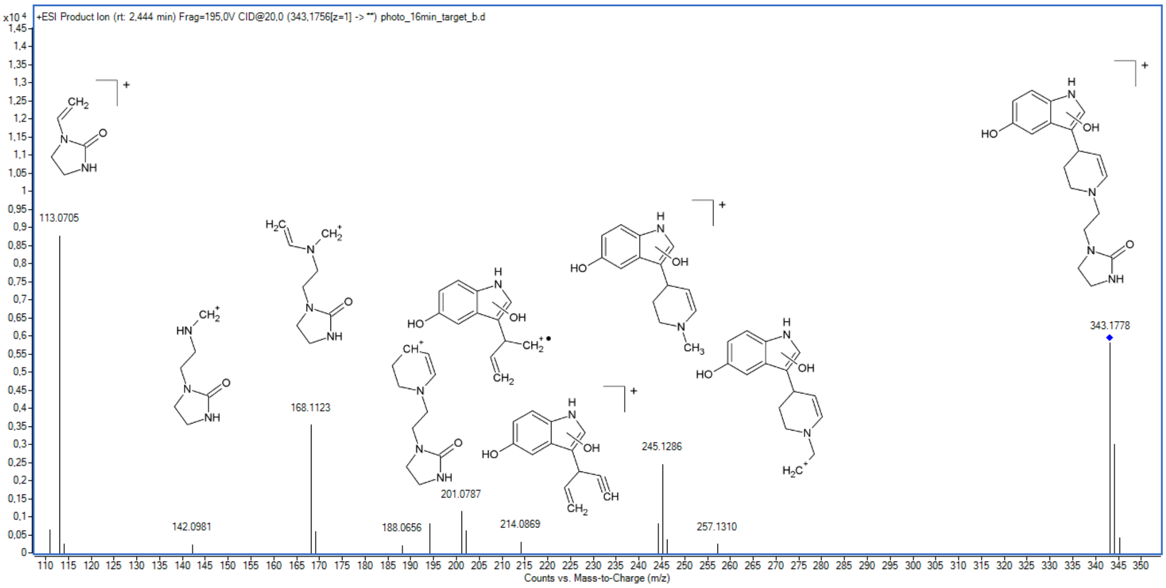

Figure S8. MS/MS spectrum and fragmentation pattern of TP7.

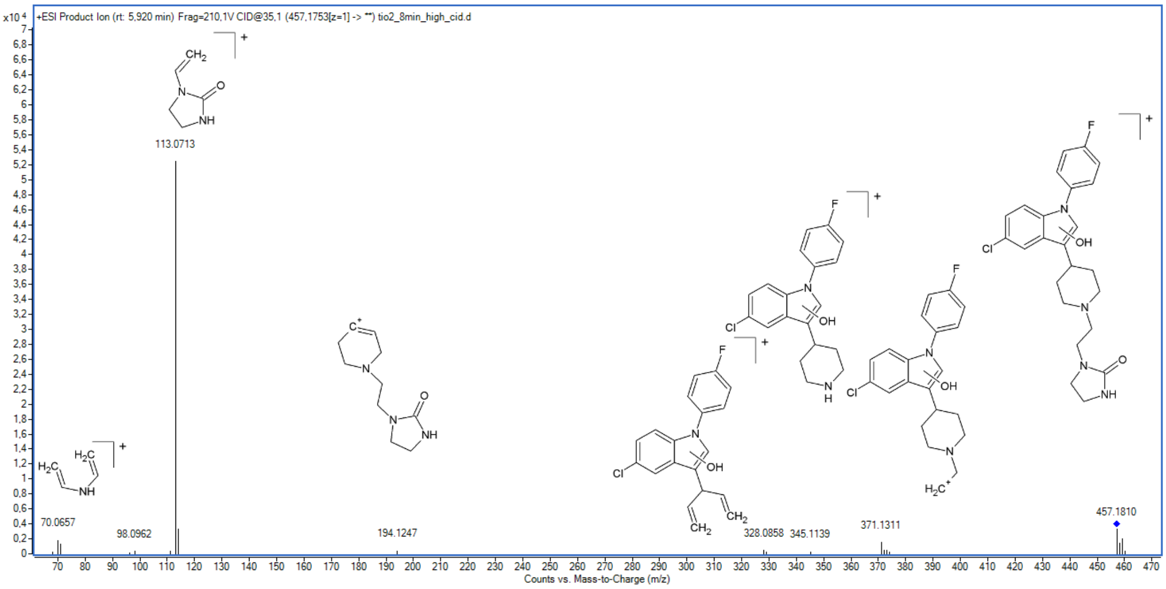

Figure S9. MS/MS spectrum and fragmentation pattern of TP8.

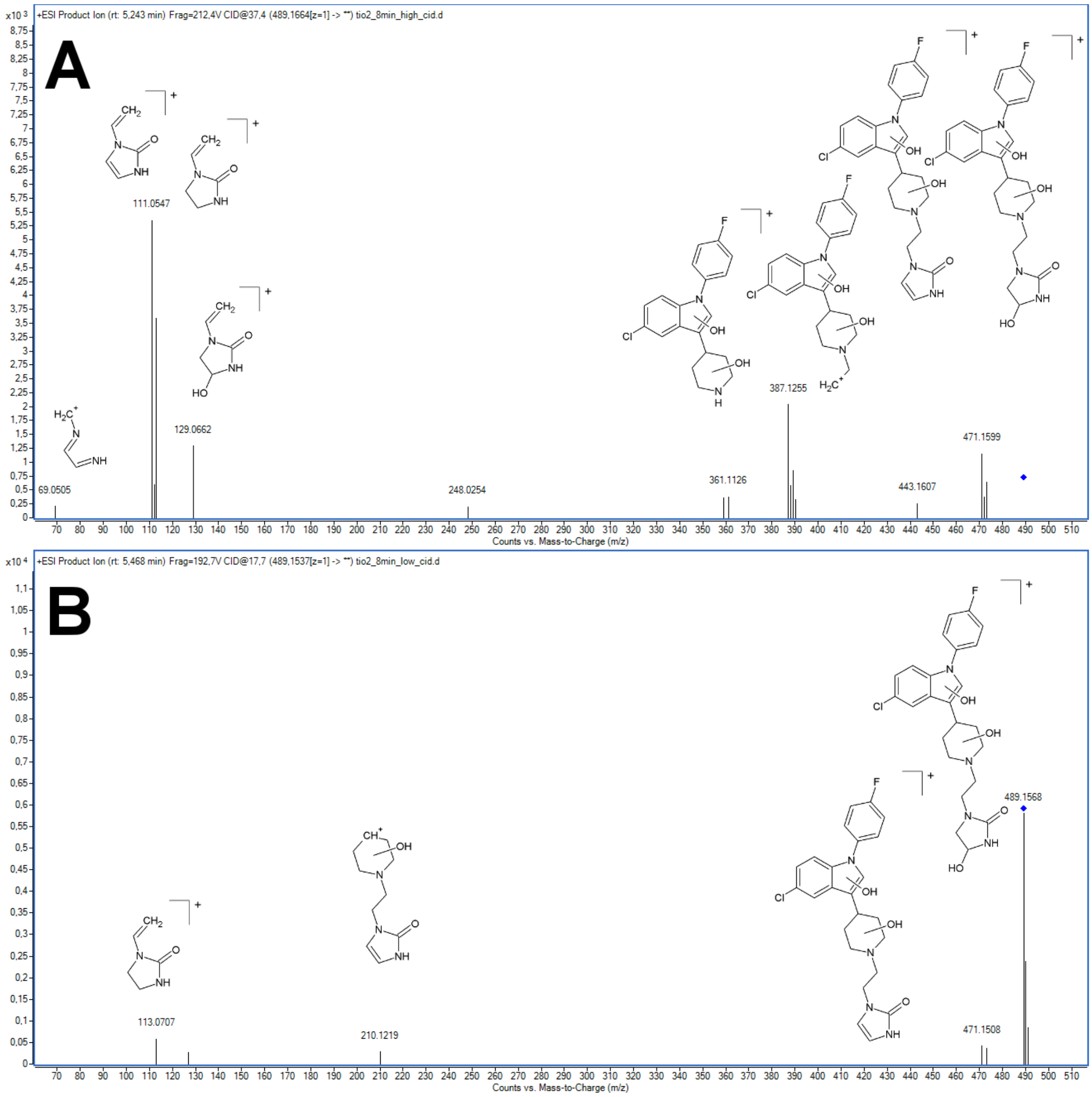

Figure S10. MS/MS spectrum and fragmentation pattern of TP9.

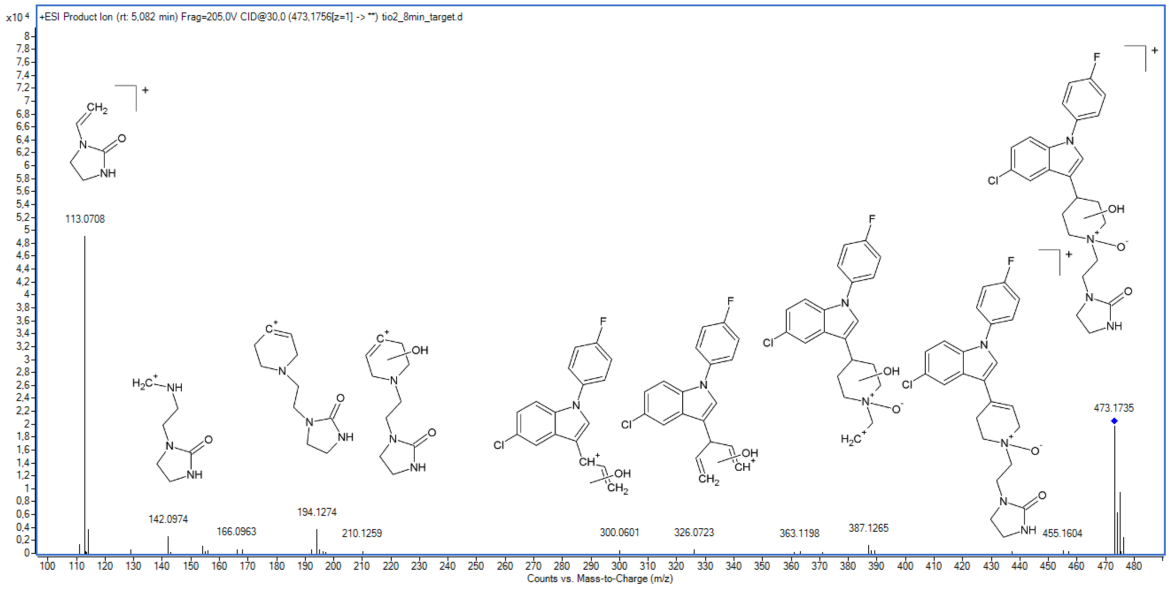

Figure S11. MS/MS spectrum and fragmentation pattern of TP10.

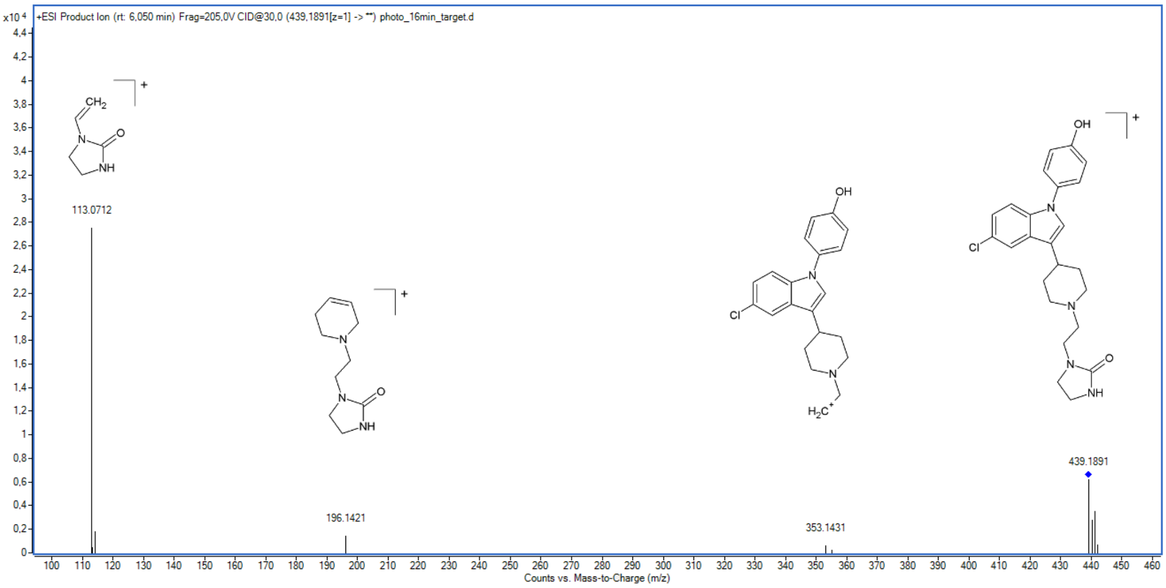

Figure S12. MS/MS spectrum and fragmentation pattern of TP11.

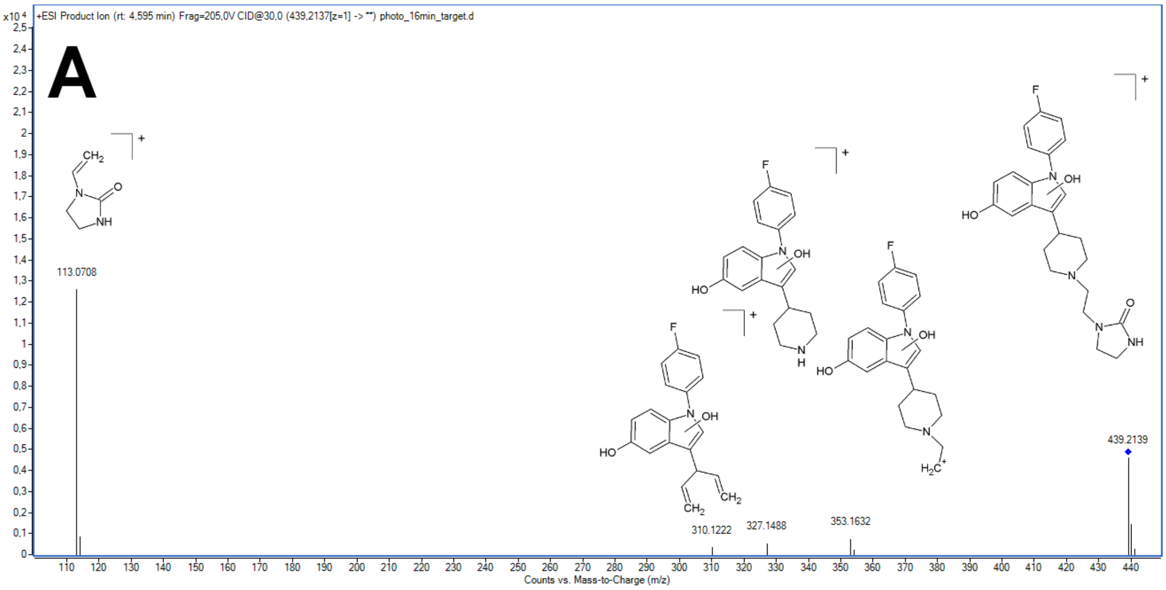

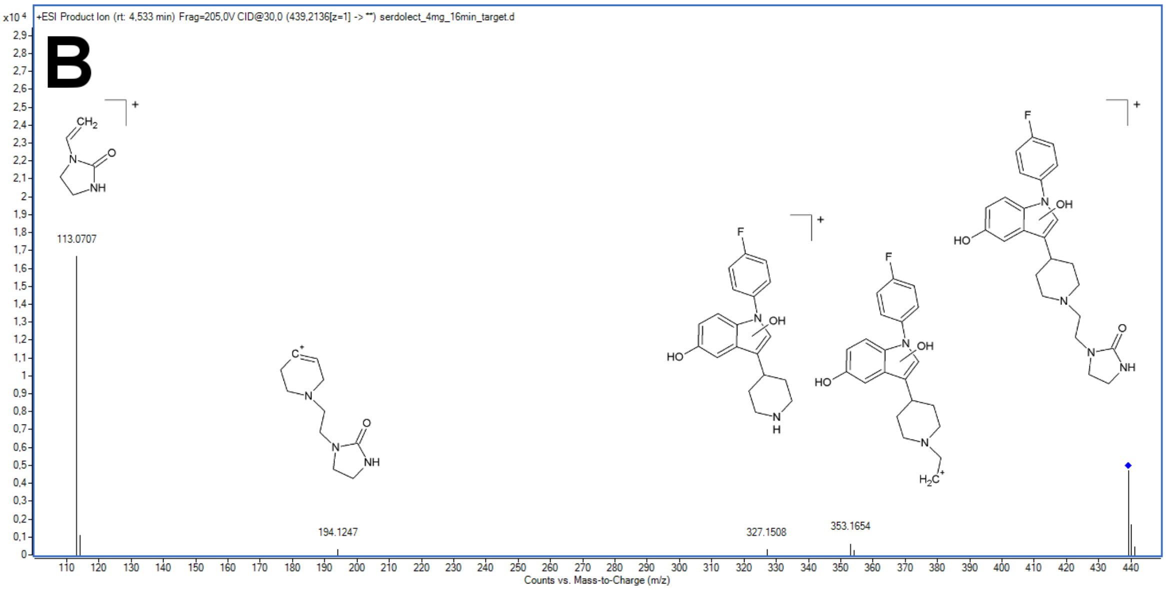

Figure S13. MS/MS spectrum and fragmentation pattern of TP12.

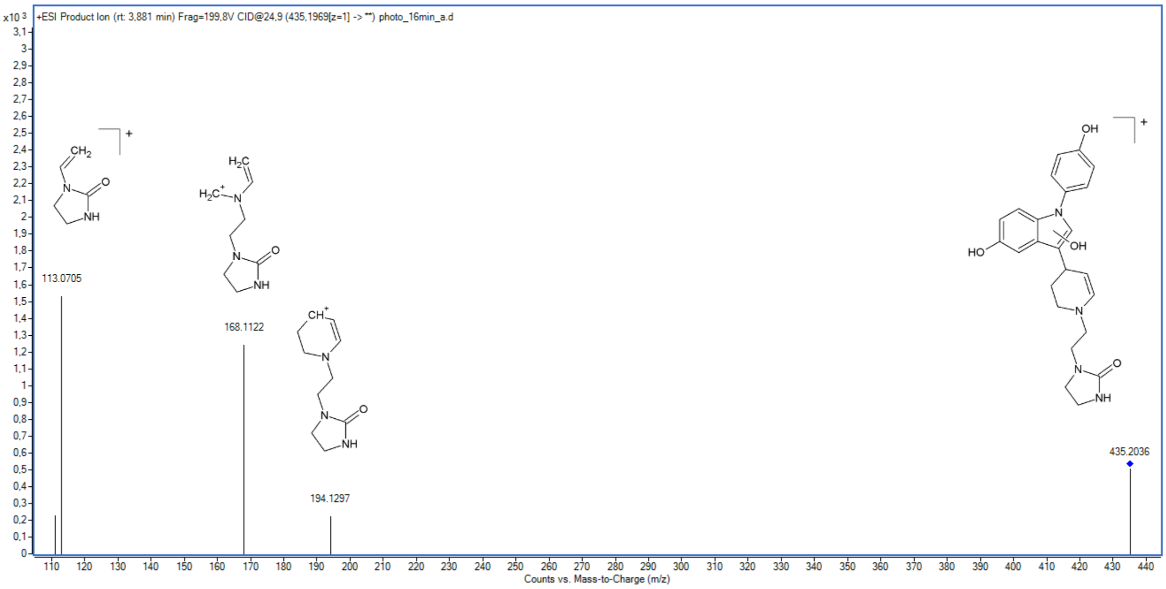

Figure S14. MS/MS spectrum and fragmentation pattern of TP13.

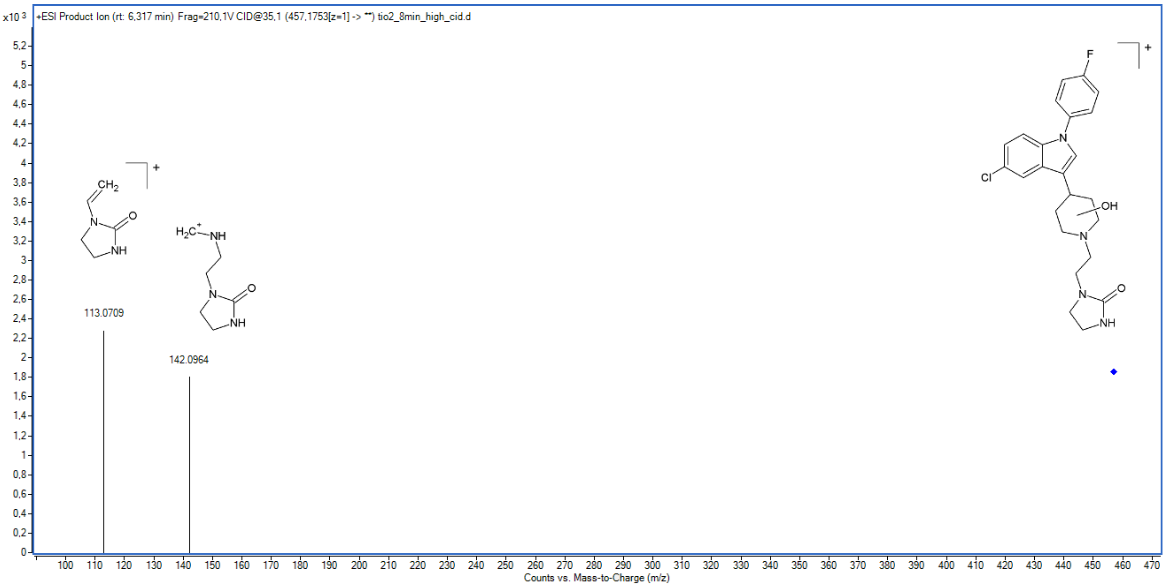

Figure S15. MS/MS spectrum and fragmentation pattern of TP14.

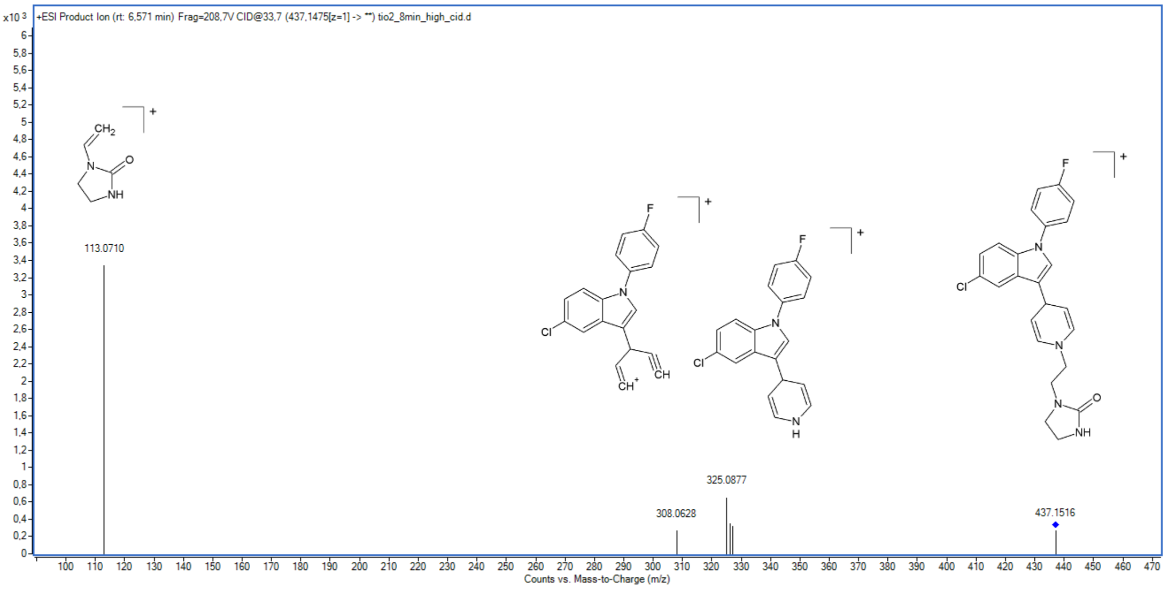

Figure S16. MS/MS spectrum and fragmentation pattern of TP15.

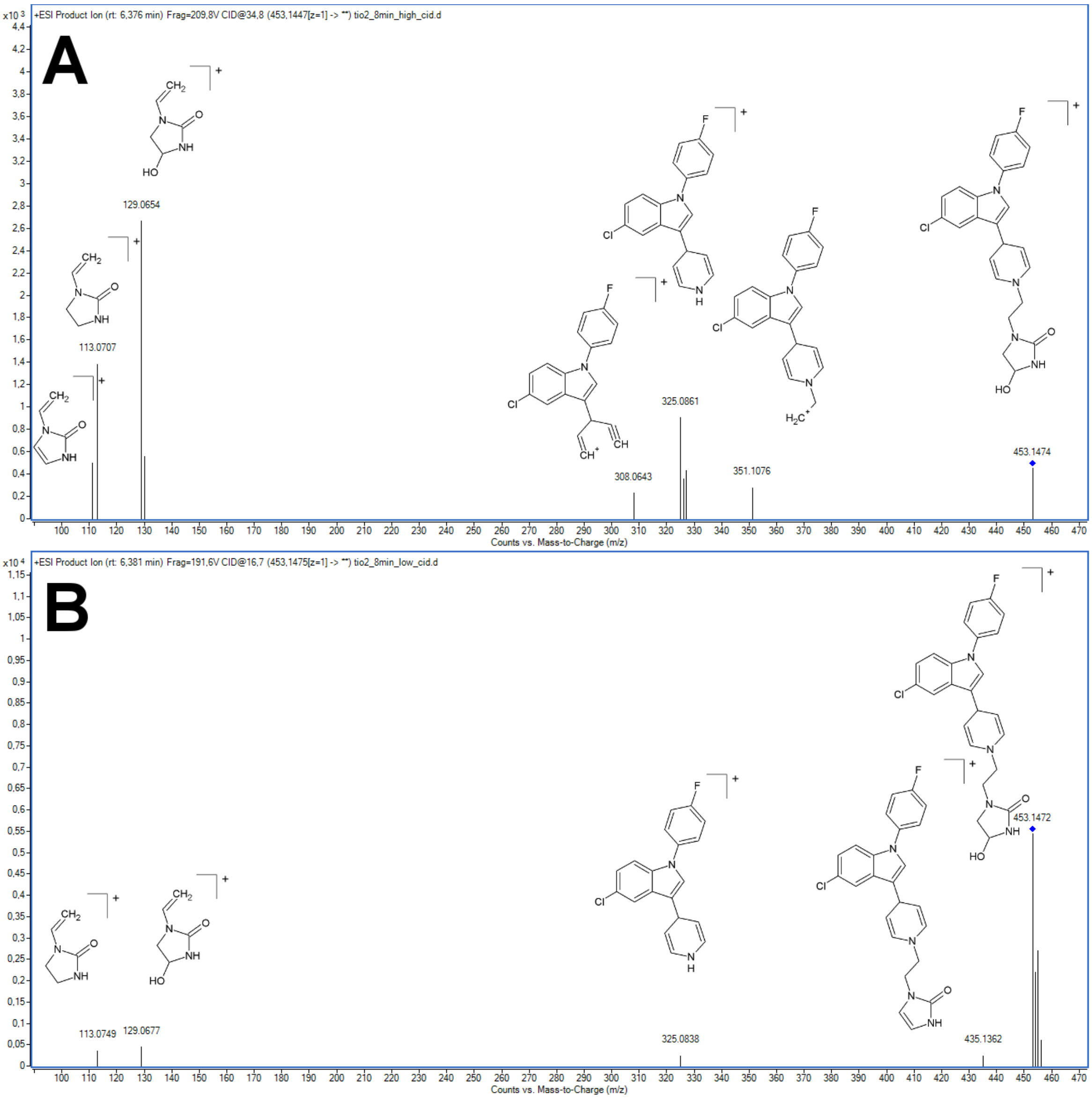

Figure S17. MS/MS spectrum and fragmentation pattern of TP16.

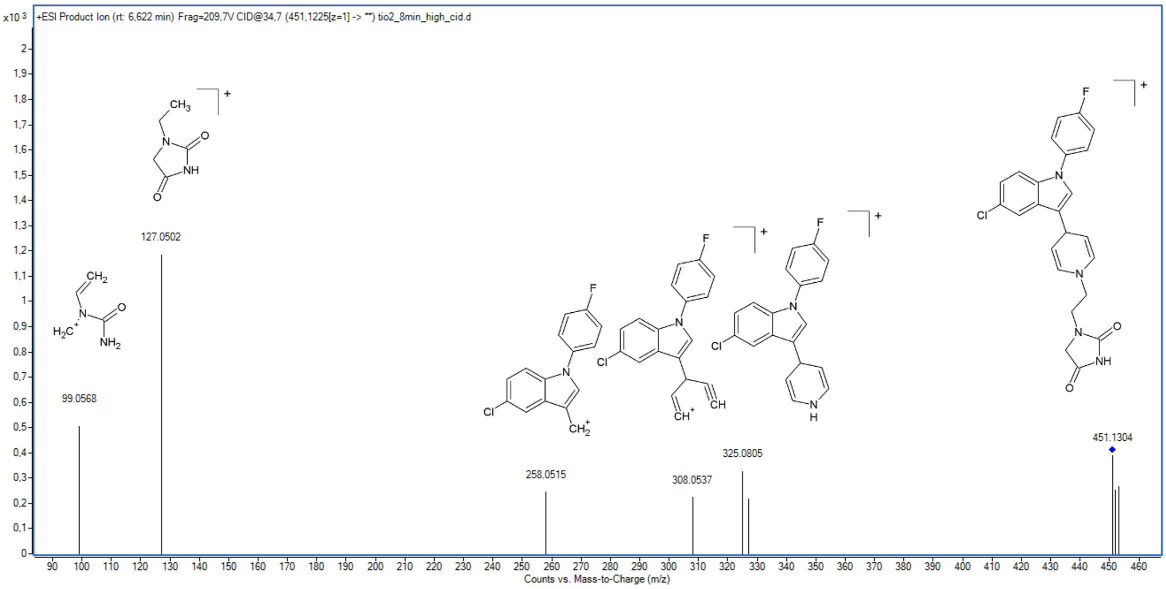

Figure S18. MS/MS spectrum and fragmentation pattern of TP17.

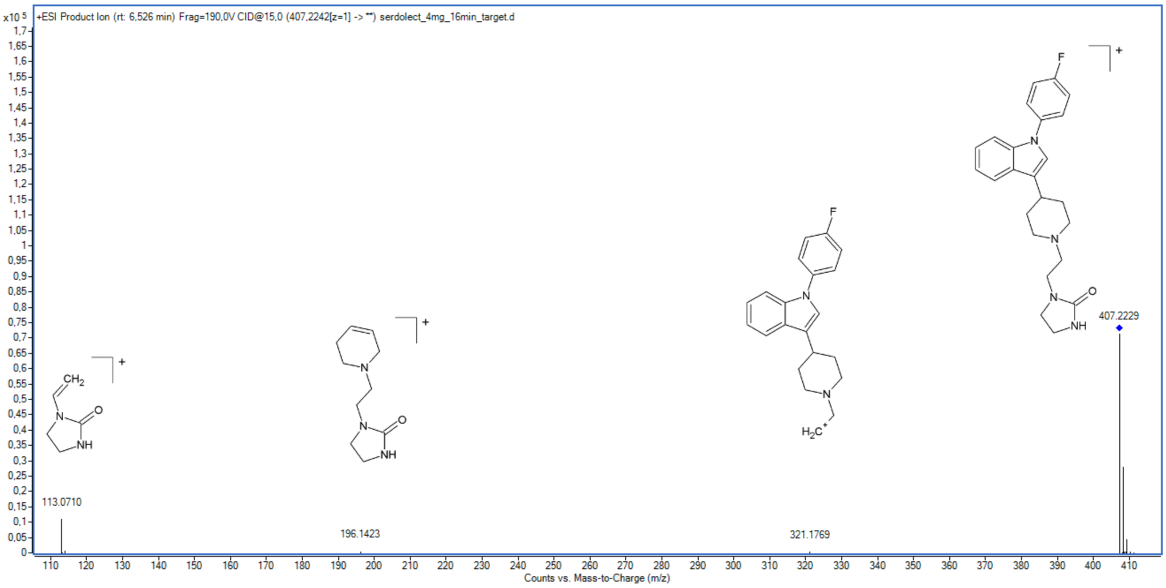

Figure S19. MS/MS spectrum and fragmentation pattern of TP18.

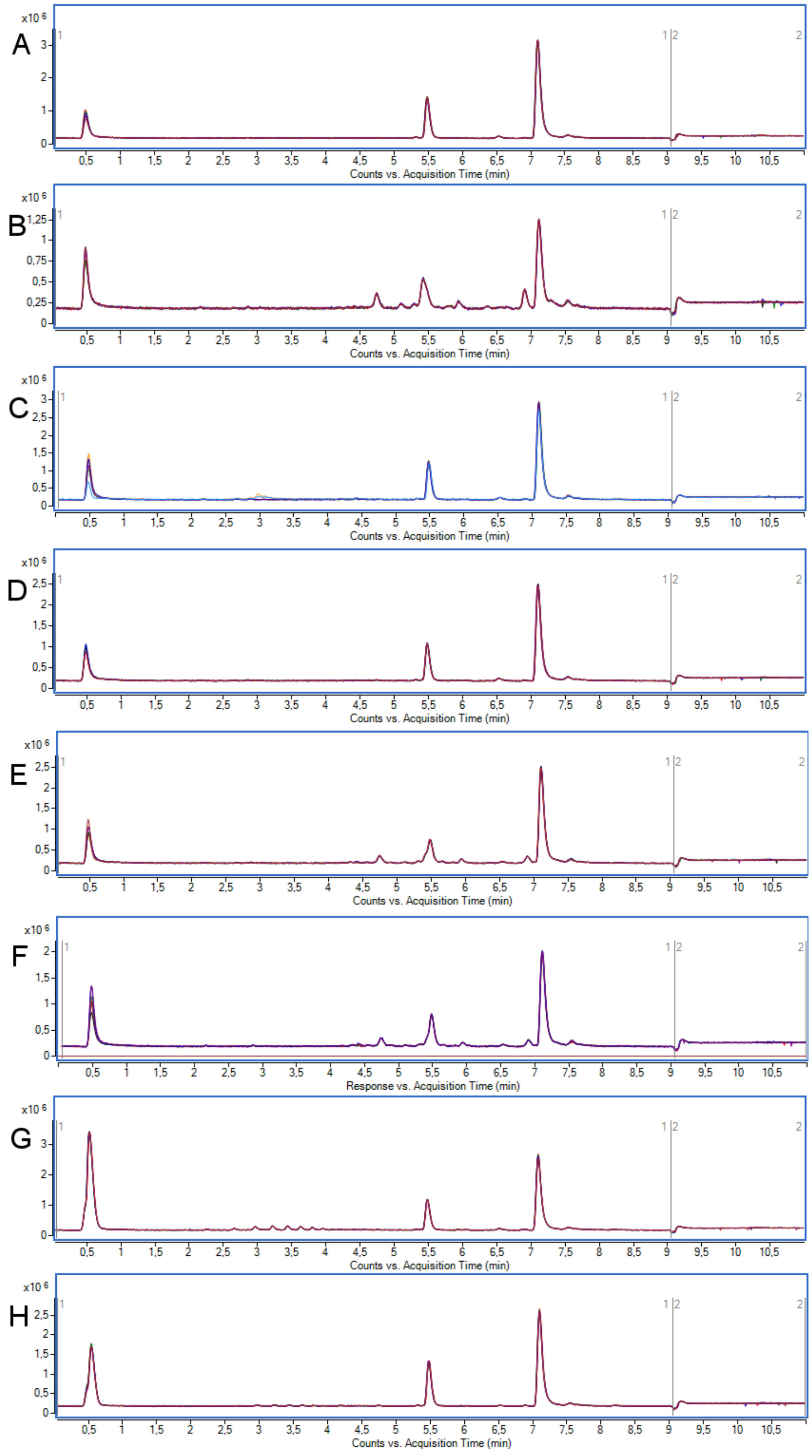

Figure S20. Total ion chromatograms (overlay) of irradiated sertindole samples (**A**: Direct photolysis, **B**: TiO_2, **C**: FeOOH yellow, **D**: Fe_2O_3 red, **E**: TiO_2- FeOOH (yellow), **F**: TiO_2- Fe_2O_3 (red), **G**: Serdolect 4 mg, **H**: Serdolect 16 mg).
